# Antimicrobial resistance of microorganisms present in periodontal diseases: A systematic review and meta-analysis

**DOI:** 10.3389/fmicb.2022.961986

**Published:** 2022-10-03

**Authors:** Flávia Casale Abe, Katia Kodaira, Cristiane de Cássia Bergamaschi Motta, Silvio Barberato-Filho, Marcus Tolentino Silva, Caio Chaves Guimarães, Carolina Castro Martins, Luciane Cruz Lopes

**Affiliations:** ^1^Department of Pharmaceutical Sciences, University of Sorocaba, Sorocaba, Brazil; ^2^Dental School, Federal University of Minas Gerais, Belo Horizonte, Brazil

**Keywords:** anti-bacterial agents, dentistry, endodontics, drug resistance, microbial, periodontics

## Abstract

**Objective:**

The aim of this study was to estimate the antimicrobial resistance in microorganisms present in periodontal diseases.

**Methods:**

A systematic review was conducted according to the PRISMA statement. The MEDLINE (PubMed/Ovid), EMBASE, BVS, CINAHL, and Web of Science databases were searched from January 2011 to December 2021 for observational studies which evaluated the antimicrobial resistance in periodontal diseases in permanent dentition. Studies that allowed the antimicrobial consumption until the time of sample collection, studies that used laboratory acquired strains, studies that only characterized the microbial strain present, assessment of cellular morphological changes, sequencing system validation, and time series were excluded. Six reviewers, working in pairs and independently, selected titles, abstracts, and full texts extracting data from all studies that met the eligibility criteria: characteristics of patients, diagnosis of infection, microbial species assessed, antimicrobial assessed, identification of resistance genes, and virulence factors. “The Joanna Briggs Institute” critical appraisal for case series was adapted to assess the risk of bias in the included studies.

**Results:**

Twenty-four studies (*N* = 2.039 patients) were included. *Prevotella* and *Porphyromonas* species were the most cited microorganisms in the included studies, and the virulence factors were related to *Staphylococcus aureus.* The antimicrobial reported with the highest frequency of resistance in the included studies was ampicillin (39.5%) and ciprofloxacin showed the lowest frequency of resistance (3.4%). The most cited genes were related to macrolides. The quality of the included studies was considered critically low.

**Conclusion:**

No evidence was found regarding the profile of antimicrobial resistance in periodontal diseases, requiring further research that should focus on regional population studies to address this issue in the era of increasing antimicrobial resistance.

**Clinical relevance:**

The knowledge about the present microorganism in periodontal diseases and their respective antimicrobial resistance profiles should guide dentists in prescribing complementary therapy for these infections.

**Systematic review registration:**

[http://dx.doi.org/10.1097/MD.0000000000013158], identifier [CRD42018077810].

## Introduction

Periodontal diseases are polymicrobial oral infections composed predominantly of species of gram-negative subgingival, capnophilic, and anaerobic bacteria. According to clinical practice, the treatment of periodontitis involves the mechanical elimination of the microbial biofilm that causes inflammation and/or infection (Skucaite et al., 2010; Ardila and Bedoya-Garcia, 2020). However, in some cases, in addition to the mechanical debridement of infected periodontal pockets, the clinical treatment protocol for severe forms of periodontitis may involve the adjuvant use of antibiotics (Kulik et al., 2019; Arredondo et al., 2020).

The microbial resistance assessment that uses disk diffusion is one of the oldest approaches and remains one of the most widely used antimicrobial susceptibility testing methods in routine clinical laboratories (EUCAST, 2022). The method of microbial identification culture-dependent is carried out by supplying the necessary nutrients and appropriate physicochemical conditions for the propagation of microorganisms in the laboratory.

Nonetheless, because not all microorganisms are cultivable under artificial conditions, culture-independent molecular biology methods have been increasingly used over the last decades. The molecular microbial identification method (non-culture dependent) relies on certain genes that contain information about the microbial identity and provides reliability for the assumed phylogenetic relationships (Munson et al., 2004).

The knowledge of the most common pathogens associated with periodontal abscess and their susceptibility profiles is necessary for a rational antimicrobial prescription (Irshad et al., 2020). The inappropriate use of antibiotics can lead not only to increased adverse events and healthcare costs but also to the risk of developing methicillin-resistant *Staphylococcus aureus* (MRSA), vancomycin-resistant enterococci (VRE), and multidrug-resistant (MDR) of gram-negative bacteria (Tacconelli et al., 2016).

The indiscriminate prescribing of antimicrobials indicates a negative contribution of health professionals to antimicrobial resistance and shows the scarcity of knowledge between the public availability of antimicrobials without prescription, and the remaining use of antimicrobials; which is the hallmark of low- and middle-income countries (Alzahrani et al., 2020).

Furthermore, resistance genes can easily spread under natural conditions. This is consistent with the rapid emergence of resistance in the clinic and predicts that new antibiotics will be selected for pre-existing determinants of resistance that have been circulating within the microbial pan-genome for millennia (D’Costa et al., 2011).

The dearth of syntheses that estimate and describe the profile of microbial resistance to antimicrobials (antibiotics and antifungals) in periodontal diseases led to the development of this systematic review. Therefore, this systematic review aimed to answer the focused question “What is the antimicrobial resistance profile of microorganisms in periodontal diseases?”, limiting the search for studies carried out from January 2011 to December 2021.

## Materials and methods

This systematic review followed Preferred Reporting Items for Systematic reviews and Meta-Analyses (PRISMA) recommendations, and was registered on the PROSPERO database under number CRD42018077810 and the protocol was published in *Medicine* (Abe et al., 2018).

### Search strategy

The search was oriented by an experienced librarian in the following databases: Medline, Embase, BVS^1^, CINAHL, and Web of Science. Additionally, the website “bancodeteses.capes.gov.br” and Gray Literature Report were searched as gray literature.

The electronic search strategy was developed using the keywords combining Medical Subject Heading (MeSH) terms. The Boolean operators “AND” and “OR” were applied to combine the terms and create a search strategy. The search strategies for each database and the following findings are summarized in Supplementary material 1. All articles selected were imported into the EndNote X9 (Clarivate, London, UK) reference manager to catalog the references and to facilitate the exclusion of duplicates.

### Eligibility criteria

The studies were selected according to the following inclusion criteria:

• Population (P): patients with gingivitis and have periodontal pockets or insertion-loss more than 3 mm, diagnosed with periodontal disease (aggressive, severe, chronic) based on clinical examination findings, diagnostic test results, or according to the definitions used by researchers to enroll participants in their studies, regardless of severity.
• Outcome (O): antimicrobial resistance (against antibiotics and antifungals) reported through minimal inhibitory concentration, zone of inhibition, and/or detection of resistance genes by culture-independent molecular techniques.
• Study design (S): observational studies.
• Timing: study published from January 2011 to December 2021.
• Language: no restriction.

Studies that allowed the antimicrobial consumption until the time of sample collection; studies that used laboratory acquired strains; studies that only characterized the microbial strain present or assessed the cellular morphological changes or did the sequencing system validation; time series and methodological studies, were excluded.

### Selection of studies

Six reviewers working in pairs and independently (CM, Juliana Pedroso Moraes Vilela de Castro (JPMVC), KK, SF, CM, CG) screened titles and abstracts. The same reviewers were calibrated for each step of the process (assessed the eligibility of each full-text article, data extraction, and risk of bias assessment of a determined number of studies with different quality levels). Disagreements were solved by consensus or a with the participation of a third reviewer (LL).

### Data extraction

The information was entered into an Excel spreadsheet using a predefined data collection form and the same groups of independent reviewers extracted the data.

The following data were extracted from each study: author (year)/country, duration of the study, place of recruitment, characteristics of patients (age/sex), diagnosis of infection, microbial species assessed, method of identification of microorganisms, antimicrobials assessed, identification of resistance genes and virulence factors, and conflict of interest.

### Data synthesis and statistical analysis

Due to the variety of resistances observed, the studies were grouped by antibiotic and antifungal analyzed: amikacin, amoxicillin, amoxicillin + clavulanic acid, amoxicillin + metronidazole, ampicillin, amphotericin B, azithromycin, cefazolin, cefepime, cefixime, cefotaxime, cefuroxime, cephalothin, ceftazidime, ceftriaxone, chloramphenicol, ciprofloxacin, clarithromycin, clindamycin, dicloxacillin, doxycycline, erythromycin, fluconazole, gentamicin, imipenem, itraconazole, kanamycin, levofloxacin, meropenem, metronidazole, miconazole, moxifloxacin, nystatin, ofloxacin, pefloxacin, penicillin, quinupristin/dalfopristin, rifampicin, roxithromycin, spiramycin, teicoplanin, tetracycline, tinidazole, trimethoprim-sulfamethoxazole (cotrimoxazole), and voriconazole.

Thus, it was decided to group the reports of resistance, regardless of the strain detected in the included studies. In each study, it was identified the number of resistant strains out of the total investigated strains to calculate the percentage of resistance. As the resistance percentages are highly variable, a random effects model was chosen to group proportions in meta-analyses. To stabilize the variances, the Freeman–Tukey double arc sine transformation was used and 95% confidence intervals were considered Wilson scores (Nyaga et al., 2014). Heterogeneity (*I*^2^) was calculated from the inverse variance model in a fixed-effect model. The limits of *I*^2^ > 50% to consider heterogeneous were adopted. All analyzes were performed on Stata SE 14.2 (StataCorp, College Station, TX, USA).

### Quality assessment and strength of evidence

The methodology used to assess the quality of the study was the checklist for case series from the Joanna Briggs Institute Critical Appraisal tools (Moola et al., 2017) and adapted to the research question of this systematic review. The risk of bias domains included was (1) selection bias (1.1 – inclusion criteria, 1.2 – diagnostic criteria, 1.3 – consecutive inclusion of patients, 1.4 – demographic characteristics of patients, 1.5 – clinical information of patients, 1.6 – location of the recruitment, and 1.7 – complete inclusion of patients); (2) measurement bias (sample collection methodology); and (3) selective reporting bias (outcome reporting). Each domain or subdomain was assessed as follows:

#### Domain 1: Selection bias

##### Inclusion criteria

*“Were the inclusion criteria clearly defined?”* The authors should provide the inclusion criteria (and exclusion if applicable) of the patients included in the studies. The inclusion criteria should present the diagnosis of the disease and the patient’s medical history.

##### Diagnostic criteria

*“Was the disease diagnostic established following a standardized criterion? The same criterion was used for all patients?” The authors should describe the measurement method of the condition (diagnostic) following a standard that should be replicated.*

##### Consecutive inclusion of patients

*“Did the study present the consecutive inclusion of patients?”* Studies that present the consecutive inclusion of patients are more reliable than those which do not present it. The authors should report if the inclusion of patients was consecutive or the period of time in which the samples were collected.

##### Demographic characteristics of patients

*“Were the demographic characteristics of patients clearly reported?”* The authors should describe the demographic characteristics of patients, such as ethnicity, sex, age, oral hygiene habits, and regional human development index (HDI).

##### Clinical information of patients

*“Was the clinical information of patients clearly reported?”* The authors should clearly report the clinical information of patients, as the condition and stage of the disease, comorbidities, harmful habits (such as smoking and drinking alcohol), etc.

##### Location of the recruitment

*“Was the location of the recruitment the same for all sample collection?”* Some diseases or conditions may vary their prevalence according to the different geographical regions and/or populations.

##### Complete inclusion of patients

*“Did the authors report the complete inclusion of the patients?”* The integrity of a case series contributes to its reliability. The authors should report if there was a loss of sample/participant and how it was solved.

#### Domain 2: Measurement bias (sample collection methodology)

“*Was the sample collection methodology adequate and standardized to all patients?”* The authors should determine if the measurement tools used were validated instruments as they have a significant impact on the validity of the outcome assessment.

#### Domain 3: Selective reporting bias (outcome reporting)

*“Were the outcome or follow-up results (microbial resistance) clearly reported?”* The results of any intervention or treatment should be clearly reported by the authors.

The Grading of Recommendations, Assessment, Development, and Evaluations (GRADE) approach framework is used to assess the certainty of evidence when data are narratively summarized (Balshem et al., 2011). Under this approach, high certainty in the evidence means that researchers are very confident that the effect they found in the studies is close to the true effect, low certainty of evidence means that the result obtained will most likely be sufficiently different from what the research has found to affect a decision, and very low certainty of evidence means that the authors are certain they have little or no confidence in the effect.

Considering these aspects, an adaptation was performed, judging the quality based only on the risk of bias. In this way, each domain was evaluated and items 1.3, 1.4, 1.5, 1.7, and 2 were used as critical domains. Observational studies, according to GRADE (Balshem et al., 2011), initially present low quality; however, if the study does not present criteria that lower its quality, it can raise a level of confidence, being considered as “moderate” quality. That is, if the study presents “low risk of bias” in all items evaluated, the confidence in this study will be considered “moderate.” If the study presents “high risk of bias” in only one critical domain or in more than one non-critical domain, trust in that study will be considered “low.” If the study presents “high risk of bias” in more than one domain considered critical, then confidence in the study will be “critically low.”

## Results

### Study selection

**FIGURE 1:**
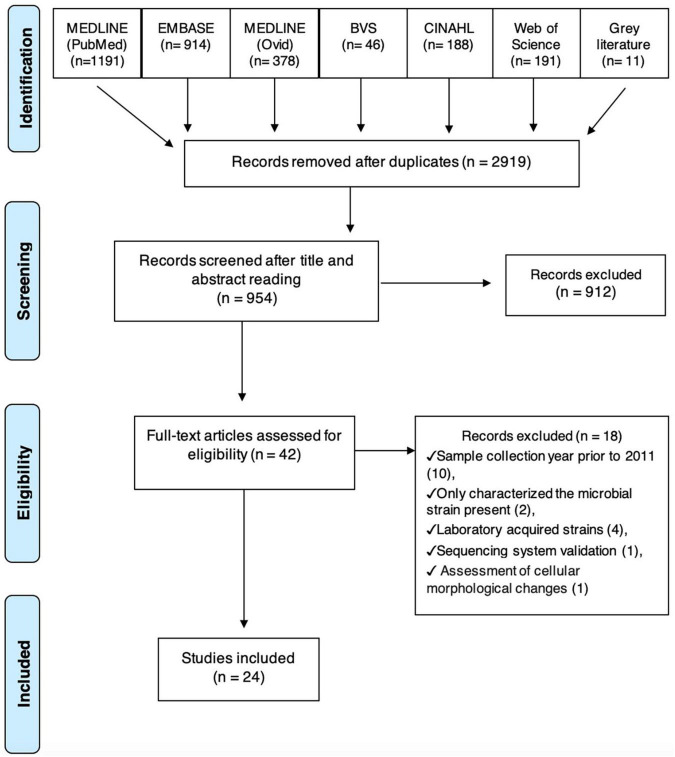
Flow chart.

The search strategy identified 2.919 titles and abstracts from the cited databases and 954 studies were screened. From the evaluation of the full text of each article, 42 studies were selected for data extraction and 24 studies met the eligibility criteria and composed the final sample used in the qualitative and quantitative analyses (Figure 1). Eighteen studies were excluded for the reasons listed in the table of characteristics of excluded studies (Supplementary material 2).

### Data extraction

Supplementary material 3 presents the characteristics of the included studies. The main findings were summarized as follows.

#### Species assessed

Thirty-nine species of microorganisms evaluated were present in periodontal pockets and/or gingival sulcus of 2.039 patients diagnosed with periodontitis (aggressive, chronic, or non-specific).

#### Country/continent

The majority of the studies were conducted in the Americas (12 studies), followed by Asia and Europe (five studies each), and Africa (two studies).

#### Study design

All of the studies were case series. Other observational studies as cohort or cross-sectional studies were not found.

#### Duration of the study

Nine studies reported the duration of the study (Gamboa et al., 2013; Xie et al., 2014; Binta and Patel, 2016; Arredondo et al., 2019, 2020; Mínguez et al., 2019; Almeida et al., 2020; Ardila and Bedoya-Garcia, 2020; Irshad et al., 2020).

#### Place of recruitment

Fifteen studies (Gamboa et al., 2013, 2014; Ehrmann et al., 2014; Xie et al., 2014; Collins et al., 2016; Bhardwaj et al., 2017; De-la-Torre et al., 2017; Arredondo et al., 2019, 2020; Bhat et al., 2019; Mínguez et al., 2019; Almeida et al., 2020; Ardila and Bedoya-Garcia, 2020; Irshad et al., 2020; Aguilar-Luis et al., 2021) were conducted in a University/Dentistry College. Three studies were conducted in a public dental clinic (Binta and Patel, 2016; Akrivopoulou et al., 2017; Ansiliero et al., 2021), and other three studies were conducted in private clinics (Rams et al., 2011, 2014b,^2020^). Two studies reported the association of more than one type of establishment (Rams et al., 2014a; Uribe-Garcia et al., 2019), and one study did not report the place of recruitment (Dhotre et al., 2015).

#### Characteristics of the patients

##### Age

Eighteen studies reported the participants’ age, ranging from 16 to 83 years.

##### Sex

The 19 studies that reported the patients’ sex, observed the feminine majority.

#### Diagnosis of the disease

Three studies reported chronic periodontal infection (Gamboa et al., 2014; Bhardwaj et al., 2017; De-la-Torre et al., 2017), two studies reported a diagnosis of aggressive infection (Akrivopoulou et al., 2017), six studies reported a diagnosis of severe/moderate infections (Rams et al., 2011, 2014a,2014b,^2020^; Binta and Patel, 2016; Bhat et al., 2019), two studies reported more than one type of infection (Collins et al., 2016; Arredondo et al., 2020), and 11 studies did not report the diagnosis of periodontal infection (Gamboa et al., 2013; Ehrmann et al., 2014; Xie et al., 2014; Dhotre et al., 2015; Arredondo et al., 2019; Mínguez et al., 2019; Uribe-Garcia et al., 2019; Almeida et al., 2020; Irshad et al., 2020; Aguilar-Luis et al., 2021; Ansiliero et al., 2021).

#### Microbial species assessed

Thirty-nine species were assessed (*Actinomyces* spp., *Aggregatibacter actinomycetemcomitans, Alloprevotella* spp., *Anaerococcus* spp., Bacteroides, *Bifidobacterium* spp., *Campylobacter* spp., *Candida* spp., *Capnocytophaga* spp., *Citrobacter freundii, Clostridium* spp., *Dialister* spp., *Eikenella* spp., Enteric rods/pseudomonads, *Enterobacter* spp., *Enterococcus* spp., *Escherichia* spp., *Erwinia* spp., *Fusobacterium* spp*., Granulicatella* spp*., Hafnia alvei, Klebsiella* spp*., Leptotrichia* spp., *Morganella* spp., *Olsenella* spp., *Parvimonas micra, Peptostreptococcus* spp., *Porphyromonas* spp., *Prevotella* spp., *Propionobacterium* spp., *Pseudomonas* spp., *Raoultella* spp., *Rothia* spp., *Serratia* spp., *Shigella* spp., *Staphylococcus* spp., *Streptococcus* spp., *Tannerella forsythia*, and *Veillonella* spp.).

#### Method of identification of the microorganisms

Sixteen studies reported culture-dependent technique, two studies reported molecular culture-not dependent, and six studies reported both techniques.

#### Antimicrobials assessed

Forty-seven antimicrobials were assessed (amikacin, amifloxacin, amoxicillin, amoxicillin + clavulanic acid, ampicillin, amphotericin B, azithromycin, cefazolin, cefepime, cefixime, cefotaxime, cefuroxime, cephalothin, ceftazidime, ceftriaxone, chloramphenicol, ciprofloxacin, clarithromycin, clindamycin, dicloxacillin, doxycycline, erythromycin, fosfomycin, fluconazole, gentamicin, imipenem, itraconazole, kanamycin, levofloxacin, linezolid, meropenem, metronidazole, miconazole, moxifloxacin, nystatin, ofloxacin, pefloxacin, penicillin, quinupristin/dalfopristin, rifampicin, roxithromycin, spiramycin, teicoplanin, tetracycline, tinidazole, trimethoprim-sulfamethoxazole (cotrimoxazole), and voriconazole).

#### Resistance genes and/or virulence factors identified

Thirty-five genes and/or virulence factors were assessed (*_*bla*_*CblA, *_*bla*_*CepA, *_*bla*_*CfxA, *_*bla*_*CSP-1, *_*bla*_*SHV, *_*bla*_*TEM, *_*bla*_*cfxA2/*_*bla*_*cfxA3/*_*bla*_*cfxA6, *tet*, *tet*B, *tet*L, *tet*M, *tet*Q, *tet*O, *tet*W, *tet*Q, *erm*(B), *erm*(C), *erm*(F), *nim*, aac, bbp, clfA, clfB, cna, coa, ebps, fnbA, fnbB, map/eap, mecA, pbp2b, sdrC, sdrD, sdrE, and spa).

#### Conflict of interest

**FIGURE 2:**
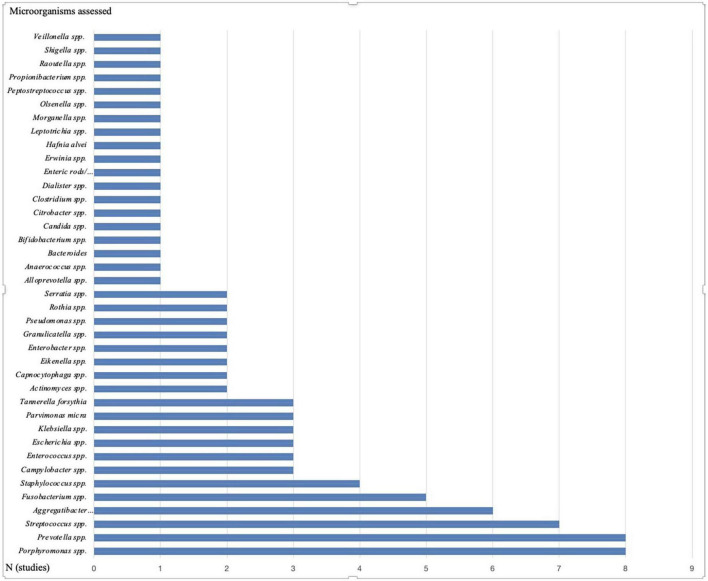
Microorganisms cited in the included studies. This figure shows the number of studies that evaluated each of the microorganisms.

*Prevotella* and *Porphyromonas* species were the most cited microorganisms in the included studies, being reported in eight studies each, followed by *Streptococcus* spp. cited in seven studies, *Aggregatibacter actinomycetemcomitans* cited in six studies, and *Fusobacterium* spp. cited in five studies (Figure 2).

Among the most prescribed antimicrobials in dentistry, after compiling data on the frequency of antimicrobial resistance and number of studies, the most evaluated were amoxicillin, metronidazole, clindamycin, azithromycin, tetracycline, cefotaxime, amoxicillin + clavulanic acid, ciprofloxacin, penicillin, doxycycline, and ampicillin. The antimicrobials that showed the highest frequency of resistance were ampicillin (40.1%) and amoxicillin + clavulanic acid (38.4%) and the antimicrobials that showed the lowest frequency of resistance were ciprofloxacin (3.4%) and tetracycline (6.0%) (Table 1).

**TABLE 1:** Antimicrobial resistance reported in periodontal diseases according to the studies.

Antimicrobial	*N* (studies)	*R*% (95% CI)	*I*^2^ (%, p)
Ampicillin	5	40.1 (16.8, 65.4)	92.3 (*p* = 0.0)
Doxycycline	5	21.0 (11.8, 31.6)	94.5 (*p* = 0.0)
Penicillin	5	14.4 (0.0, 42.4)	95.4 (*p* = 0.0)
Ciprofloxacin	5	3.4 (0.0, 15.3)	67.2 (*p* = 0.0)
Amoxicillin + clavulanic acid	6	38.4 (17.0, 61.8)	85.7 (*p* = 0.0)
Cefotaxime	7	23.0 (10.8, 37.1)	85.3 (*p* = 0.0)
Tetracycline	8	6.0 (0.3, 15.8)	71.2 (*p* = 0.0)
Azithromycin	9	28.1 (17.0, 40.2)	78.6 (*p* = 0.0)
Clindamycin	10	28.4 (15.0, 43.5)	95.8 (*p* = 0.0)
Metronidazole	12	21.6 (10.4, 35.0)	97.5 (*p* = 0.0)
Amoxicillin	15	16.4 (8.7, 25.3)	94.1 (*p* = 0.0)
*R* – antimicrobial resistance, *I*^2^ – heterogeneity			

**FIGURE 3:**
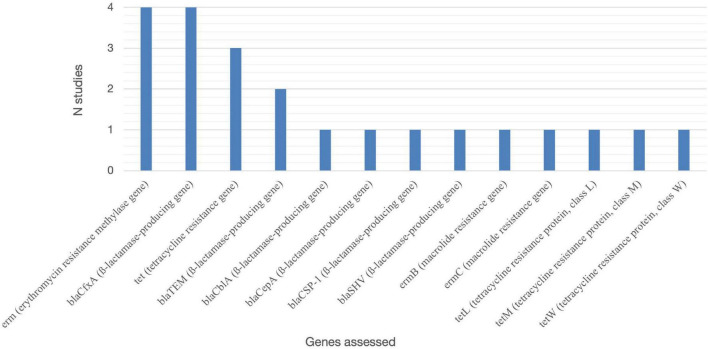
Resistance genes assessed in the included studies. erm, erythromycin resistance methylase gene; blaCfxA, ß-lactamase-producing gene; tet, tetracycline resistance gene; blaTEM, ß-lactamase-producing gene; blaCblA, ß-lactamase-producing gene; blaCepA, ß-lactamase-producing gene; blaCSP-1, ß-lactamase-producing gene; blaSHV, ß-lactamase-producing gene; ermB, macrolide resistance gene; ermC, macrolide resistance gene; tetL, tetracycline resistance protein, class L; tetM, tetracycline resistance protein, class M; tetW, tetracycline resistance protein, class W.

The most cited genes in the included studies were related to the erythromycin resistance gene (*erm*), *ß-lactamase-producing* gene (*_*bla*_cfxA*), and tetracycline resistance gene (*tet*) (Figure 3).

Only one study (Uribe-Garcia et al., 2019) cited virulence factors and they were related to *Staphylococcus aureus* and extracellular adhesion.

Five studies (Dhotre et al., 2015; Arredondo et al., 2019; Uribe-Garcia et al., 2019; Ansiliero et al., 2021) reported multidrug resistance.

The data obtained regarding the antibiotic resistance profile, the prevalence of microorganisms assessed in periodontal diseases, and the antimicrobial resistance relationship did not allow the generation of statistical analysis for this systematic review.

### Quality assessment

**FIGURE 4:**
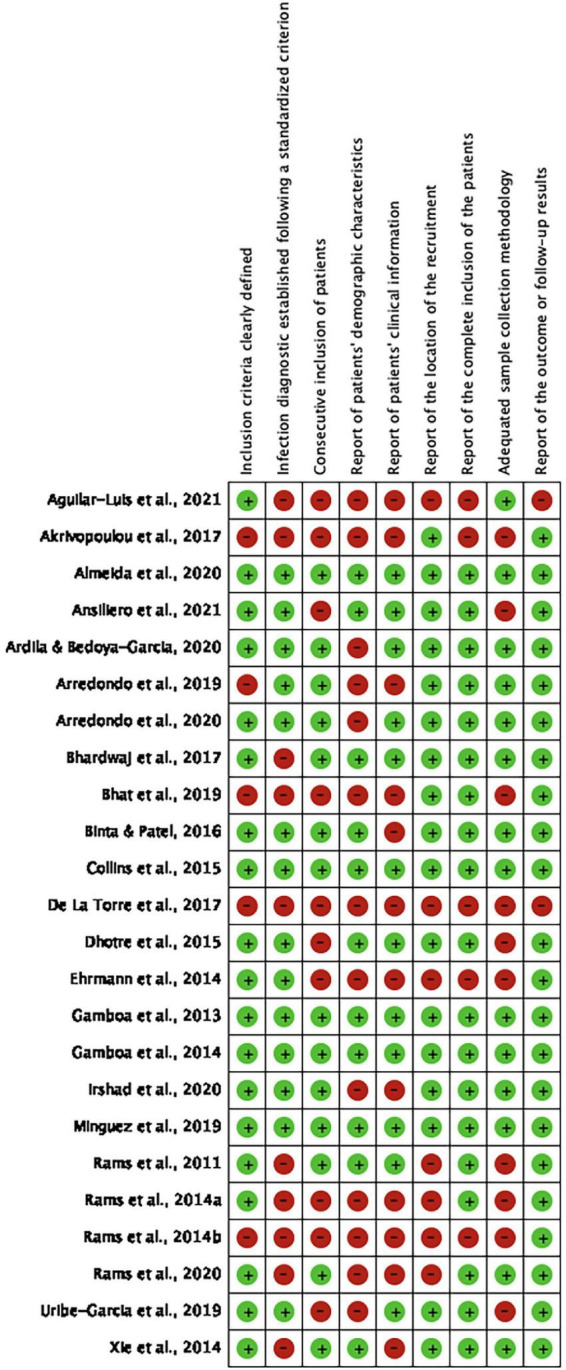
Risk of bias assessment.

Figure 4 illustrates the risk of bias in the included studies for each domain evaluated and the analysis of the overall risk of bias. Only six studies (Gamboa et al., 2013, 2014; Collins et al., 2016; Bhardwaj et al., 2017; Mínguez et al., 2019; Almeida et al., 2020) presented all the criteria evaluated as low risk of bias. The remaining 18 studies presented at least one domain considered critical as a high risk of bias; therefore, they were graded as critically low confidence.

It was not possible to analyze the risk of publication bias, as 10 studies evaluating the antimicrobial resistance of the same microorganisms against the same antimicrobials were not obtained.

## Discussion

Although the American Academy of Periodontology published in 2017 (Tonetti et al., 2018) a new classification of periodontal and peri-implant diseases and conditions, most studies included in this systematic review still use the 1999 classification.

One of the limitations of this review was the impossibility of estimating the prevalence of microorganisms found in periodontal diseases, as the studies that met the eligibility criteria were only case series presenting a lack of information on the clinical and demographic characteristics of the patients.

Another limitation of the results of this systematic review was not being possible to determine if the resistance in the microorganisms assessed is permanent or if it fades away during cultivation.

The most cited antimicrobials were not those that showed the highest rates of resistance; however, it was not possible to associate these rates with the microorganisms evaluated. Probably, the different frequencies of resistance among antimicrobials with a similar mechanism of action may be explained by the methodology for assessing antimicrobial resistance, the geographic location, and the chromosomal mutation of the population evaluated (Huttner et al., 2020).

As most of the studies included in this systematic review did not report important information about the clinical and demographic characteristics of the patients, nor the inclusion criteria of these patients in the evaluated studies, it was not possible to determine the geographic distribution of the resistant microorganisms found.

In spite of the reported microorganisms in this systematic review were present in almost all studies, it was observed that Latin American countries showed a high level of ß-lactamase, MRSA, and multidrug-resistant microorganisms (MDRO) (Ardila and Bedoya-Garcia, 2020).

Commensal bacteria present in sick and healthy patients may contribute to the development of MRSA when antimicrobials are used as adjuncts to periodontal treatment (Colombo et al., 2015). The heterogeneous etiology of periodontitis, in which multiple microbial combinations may play a role in the cause of the disease, could justify the multidrug resistance found in periodontal diseases reported in five studies included in this systematic review (Dhotre et al., 2015; Arredondo et al., 2019, 2020; Uribe-Garcia et al., 2019; Ansiliero et al., 2021).

Another factor that contributes to the increase in antimicrobial resistance is the high presence of adhesion factors that contributes to biofilm formation (Uribe-Garcia et al., 2019; Almeida et al., 2020). When microorganisms adhere to the surface of teeth, a biofilm containing numerous pathogenic species is formed as a reservoir of resistance genes, reducing treatment alternatives, and increasing microbial persistence.

The possibilities of genetic exchange and sophisticated microbial intercommunications (quorum-sensing system) lead to the horizontal transfer of genes contributing to the increase of antimicrobial resistance (Périchon and Courvalin, 2009; Paul et al., 2018). It might be supported by the hypothesis that low-level exposure to chlorhexidine, used for the control of oral biofilms, may result in the development of cross-resistance toward antibiotics (Cieplik et al., 2019).

The culture method used to determine antimicrobial resistance was one of the main limiting factors of the studies, because not all microorganisms are cultivable under artificial conditions and the *in vitro* susceptibility assessment does not accurately reflect the clinical efficacy (Munson et al., 2004; Ardila and Bedoya-Garcia, 2020).

Studies using culture-independent techniques have revealed that the oral microbiota is more diverse than previously demonstrated by culture methods and have revealed new pathogens involved in oral diseases such as caries, periodontal disease, and endodontic infections (Siqueira et al., 2012).

There is considerable uncertainty regarding microorganisms and their antimicrobial resistance in periodontal diseases; therefore, further research is needed focusing on regional population studies to resolve this problem in the era of increasing resistance to antimicrobials.

One systematic review published in 2020 (Khattri et al., 2020) assessed the effects of systematic antimicrobials as an adjunct to non-surgical periodontal treatment and concluded that there is very low-certainty evidence (for long-term follow-up) to inform clinicians and patients if adjunctive systemic antimicrobials are of any help for the non-surgical treatment of periodontitis. In addition, none of the studies reported data on antimicrobial resistance and patient reported quality of life changes.

The data reported in the included studies on the microorganisms assessed or the analysis of resistance to the antimicrobials tested presented high heterogeneity (*I*^2^) between the antimicrobials assessed. It is not possible to infer hypotheses about the resistance of the antimicrobials and their implications, nor to suggest safer and more effective therapeutic protocols.

The information collected and related in this systematic review will guide future research in order to evaluate the behavior of the microorganisms that make up the microbiome and their resistance profile to the most commonly prescribed antimicrobials in dentistry.

## Data availability statement

The original contributions presented in this study are included in the article/Supplementary material, further inquiries can be directed to the corresponding author.

## Ethics statement

Ethical review and approval was not required for the study on human participants in accordance with the local legislation and institutional requirements. Written informed consent for participation was not required for this study in accordance with the national legislation and the institutional requirements.

## Author contributions

FA was the principal investigator, wrote the protocol, and the final version. KK performed extraction of data and critique the literature and helped to write the final version. CM performed the extraction data, critique the literature, and helped to write the protocol. SB-F performed the extraction data and critique the literature and revised the manuscript. MS provided insight into the epidemiological aspects of the review and helped to draft the manuscript. CG and CCM performed the extraction data and critique the literature. LL was the review guarantor, advised on background, helped to write the protocol, and revised the manuscript. All authors contributed to the article and approved the submitted version.

## Footnotes

1
bvsalud.org
