# Supplementary material for: Antimicrobial resistance of microorganisms present in periodontal diseases: A systematic review and meta-analysis
Source: Front Microbiol. 2022 Oct 3;13:961986. doi: 10.3389/fmicb.2022.961986 (PMC9574196; doi:10.3389/fmicb.2022.961986)
Supplement: Supplementary file 1 [file Data_Sheet_1.docx]

**Supplementary file 1**. Search strategy.

Database: **PubMed**

- 1. "dental pulp cavity"[MeSH Terms]
  2. "dental care"[MeSH Terms]
  3. “root canal”[MeSH Terms]
  4. “endodontics”[MeSH Terms]
  5. “periapical abscess”[MeSH Terms]
  6. “periodontal disease”[MeSH Terms]
  7. “periododntal abscesses”[MeSH Terms]
  8. “drug resistance, microbial”[MeSH Terms]
  9. “microbial sensitivity tests”[MeSH Terms]
  10. “antifungal drug resistance”[MeSH Terms]
  11. 1 OR 2 OR 3 OR 4 OR 5 OR 6 OR 7
  12. 8 OR 9 OR 10
  13. 11 AND 12

Search ((((((((resistance, antifungal drug[MeSH Terms]) OR Antifungal Drug Resistance) OR Resistance, Antifungal Drug) OR Antibiotic Resistance, Fungal)) OR ((((((((((((((((((((((((((((microbial sensitivity test[MeSH Terms]) OR Microbial Sensitivity Test) OR Sensitivity Test, Microbial) OR Sensitivity Tests, Microbial) OR Test, Microbial Sensitivity) OR Tests, Microbial Sensitivity) OR Drug Sensitivity Assay, Microbial) OR Antimicrobial Susceptibility Breakpoint Determination) OR Breakpoint Determination, Antimicrobial Susceptibility) OR Virus Drug Sensitivity Tests) OR Viral Drug Sensitivity Tests) OR Breakpoint Determination, Antibacterial Susceptibility) OR Fungus Drug Sensitivity Tests) OR Fungal Drug Sensitivity Tests) OR Minimum Inhibitory Concentration) OR Concentrations, Minimum Inhibitory) OR Concentration, Minimum Inhibitory) OR Inhibitory Concentration, Minimum) OR Inhibitory Concentrations, Minimum) OR Minimum Inhibitory Concentrations) OR Antibiogram) OR Antibiograms) OR Bacterial Sensitivity Tests) OR Tests, Bacterial Sensitivity) OR Sensitivity Tests, Bacterial) OR Test, Bacterial Sensitivity) OR Bacterial Sensitivity Test) OR Sensitivity Test, Bacterial)) OR (((((((antibiotic resistance[MeSH Terms]) OR Drug Resistances, Microbial) OR Antimicrobial Drug Resistance) OR Antimicrobial Drug Resistances) OR Antibiotic Resistance, Microbial) OR Antibiotic Resistance) OR Resistance, Antibiotic)))

AND ((((((((((((((((((((((((((dental pulp cavity) OR cavity, dental pulp) OR pulp cavities, dental) OR pulp cavity, dental) OR cavities, dental pulp) OR dental pulp cavitites) OR dental pulp cavities) OR chamber, pulp) OR chambers, pulp) OR pulp chambers) OR pulp canal) OR canal, pulp) OR canals, pulp) OR pulp canals) OR root canal) OR canal, root) OR canals, root) OR root canals) OR pulp chamber)) OR ((dental care) OR care, dental)) OR Endodontics) OR (((((((((((((((((Pulp Canals) OR Root Canals) OR Cavity, Dental Pulp) OR Pulp Cavities, Dental) OR Pulp Cavity, Dental) OR Cavities, Dental Pulp) OR Dental Pulp Cavities) OR Pulp Chamber) OR Chamber, Pulp) OR Chambers, Pulp) OR Pulp Chambers) OR Pulp Canal) OR Canal, Pulp) OR Canals, Pulp) OR Canals, Root) OR Canal, Root) OR Root Canal)) OR (((((((((((((((((((((((Abscesses, Periapical) OR Periapical Abscesses) OR Dentoalveolar Abscess, Apical) OR Abscess, Apical Dentoalveolar) OR Abscesses, Apical Dentoalveolar) OR Apical Dentoalveolar Abscess) OR Apical Dentoalveolar Abscesses) OR Dentoalveolar Abscesses, Apical) OR Periodontitis, Apical, Suppurative) OR Periapical Periodontitis, Suppurative) OR Periapical Periodontitides, Suppurative) OR Periodontitides, Suppurative Periapical) OR Periodontitis, Suppurative Periapical) OR Suppurative Periapical Periodontitides) OR Suppurative Periapical Periodontitis) OR Alveolar Abscess, Apical) OR Abscess, Apical Alveolar) OR Abscesses, Apical Alveolar) OR Alveolar Abscesses, Apical) OR Apical Alveolar Abscess) OR Apical Alveolar Abscesses) OR Abscess, Periapical) OR periapical abscess)) OR (((((((periodontal disease[MeSH Terms]) OR Disease, Periodontal) OR Diseases, Periodontal) OR Periodontal Disease) OR Parodontosis) OR Parodontoses) OR Pyorrhea Alveolaris)) OR ((((periodontal abscess[MeSH Terms]) OR Abscess, Periodontal) OR Abscesses, Periodontal) OR Periodontal Abscesses))

Database: **Embase**

1. exp dental pulp cavity/ or dental pulp cavity.mp.
2. dental care.mp. or exp dental procedure/
3. endodontics.mp. or exp endodontics/
4. periapical abscess.mp. or exp tooth periapical disease/
5. periodontal disease.mp. or exp periodontal disease/
6. periodontal abscess.mp. or exp periodontal abscess/ or exp periodontal disease/
7. drug resistance, microbial.mp. or exp antibiotic resistance/
8. microbial sensitivity tests.mp. or exp microbial sensitivity test/
9. antifungal drug resistance.mp. or exp antifungal resistance/
10. 1 OR 2 OR 3 OR 4 OR 5 OR 6
11. 7 OR 8 OR 9
12. 10 AND 11

Database: **CINAHL**

1. (MH "Dental Pulp Cavity") OR "dental pulp cavity"
2. (MH "Dental Care+") OR "dental care"
3. “root canal" OR (MH "Root Canal Therapy")
4. (MH "Endodontics+") OR "endodontics"
5. “periapical abscess" OR (MH "Periapical Diseases")
6. “periodontal disease" OR (MH "Periodontal Diseases+")
7. (MH "Periodontal Abscess") OR "periodontal abscess"
8. (MH "Drug Resistance, Microbial+") OR "drug resistance, microbial" OR (MH "Drug Resistance+")
9. (MH "Microbial Culture and Sensitivity Tests") OR "microbial sensitivity test"
10. 1 OR 2 OR 3 OR 4 OR 5 OR 6 OR 7
11. 8 OR 9
12. 10 AND 11
