# Supplementary material for: Antimicrobial resistance of microorganisms present in periodontal diseases: A systematic review and meta-analysis
Source: Front Microbiol. 2022 Oct 3;13:961986. doi: 10.3389/fmicb.2022.961986 (PMC9574196; doi:10.3389/fmicb.2022.961986)
Supplement: Supplementary file 2 [file Data_Sheet_2.docx]

**Supplementary file 2 –** *Reasons for exclusion*

Excluded studies (n = 18)

| Author, year | Reasons for exclusion |
| --- | --- |
| Benachinmardi et al., 2013 | Sample collection year prior to 2011 |
| Benachinmardi et al., 2015 | Sample collection year prior to 2011 |
| Egwari et al., 2016 | Sample collection year prior to 2011 |
| Fernandez-Canigia et al., 2015 | Sample collection year prior to 2011 |
| He et al., 2013 | Sample collection year prior to 2011 |
| Koukos et al., 2013 | Sample collection year prior to 2011 |
| Lourenço et al., 2015 | Sample collection year prior to 2011 |
| Mombelli et al., 2015 | Sample collection year prior to 2011 |
| Oettinger-Barak et al., 2013 | Sample collection year prior to 2011 |
| Rasteniene et al., 2015 | Sample collection year prior to 2011 |
| Lee & Lee, 2019 | Laboratory acquired strains |
| Okamoto-Shibayama et al., 2017 | Laboratory acquired strains |
| Song et al., 2013 | Laboratory acquired strains |
| Tantivitayakul et al., 2020 | Laboratory acquired strains |
| Diaz et al., 2015 | Assessment of cellular morphological changes |
| Stahli et al., 2020 | Only characterized the microbial strain present |
| Koukos et al., 2015 | Only characterized the microbial strain present |
| Marin et al., 2019 | Sequencing system validation |
