# Supplementary material for: Antimicrobial resistance of microorganisms present in periodontal diseases: A systematic review and meta-analysis
Source: Front Microbiol. 2022 Oct 3;13:961986. doi: 10.3389/fmicb.2022.961986 (PMC9574196; doi:10.3389/fmicb.2022.961986)
Supplement: Supplementary file 3 [file Data_Sheet_3.docx]

**Supplementary Material 3** – Characteristics and main findings of the included studies.

| Continent (country) | Author/ year | Population (N)/ Diagnosis | Participants’ age (range) | Microbial species assessed (N) | Technique assessment | Antimicrobials assessed (*resistance) | Resistance gene (Antimicrobial) | Virulence factors (Antimicrobial) |
| --- | --- | --- | --- | --- | --- | --- | --- | --- |
| *Africa*  *(Morocco)* | Minguez *et al.,* 2019 | Patients (45)  Non-specific periodontal disease | 24,4 (mean) | *Aggregatibacter*  *actinomycetemcomitans* (24)  *Porphyromonas gingivalis* (30) | Culture | AMX, AXL, AZI*, MTZ* | NA | NA |
| *Africa*  *(South Africa)* | Binta & Patel, 2016 | Patients (48)  Severe or moderate chronic periodontitis | 52 (mean) | *Prevotella* spp. (49)  *Bacteroides* (5)  *Porphyromonas* spp. (6)  *Fusobacterium* spp. (4)  *Clostridium* spp. (3)  *Propionobacterium* spp. (3) | Culture  Molecular technique | AMP*, PEN* | *_bla_cfxA* 2/ *_bla_cfxA* 3/ *_bla_cfxA* 6 (ß-lactamase) | NA |
| *Americas*  *(United States of America)* | Rams *et al.,* 2011 | Patients (37)  Severe periodontitis | 57,5 + 12 (range) | *Porphyromonas gingivalis* (15)  *Tannerella forsythia* (19)  *Prevotella intermedia*/  *nigrescens* (36)  *Fusobacterium nucleatum* (36)  *Parvimonas micra* (37)  *Campylobacter rectus* (20)  *Streptococcus constellatus* (10)  *Streptococcus intermedius* (10) | Culture | AMX*, SPI*, MTZ*  AMX + MTZ*, SPI + MTZ* | NA | NA |
| *Americas*  *(Colombia)* | Gamboa *et al.,*2013 | Patients (86)  Non-specific periodontal disease | >20 years | *Klebsiella pneumoniae* (4)  *Klebsiella oxytoca* (5)  *Escherichia coli* (3)  *Hafnia alvei* (2)  *Erwinia* spp. (3)  *Shigella* spp. (2)  *Serratia liquefaciens* (5)  *Serratia marcescens* (2)  *Serratia odorifera* (1)  *Enterobacter cloacae* (2) | Culture | AMI*, AXL*, AMP*, KAN*, CFM*, CZD*, CXE*, CIP*, CTZ*, GEN* | NA | NA |
| *Americas*  *(Colombia)* | Gamboa *et al.,*2014 | Patients (87)  Chronic periodontal disease | NR | *Porphyromonas gingivalis* (30) | Culture | MTZ, TET* | NA | NA |
| *Americas*  *(United States of America)* | Rams *et al.,* 2014a | Patients (400)  Severe periodontal disease | 35 – 78 years | *Porphyromonas gingivalis* (312)  *Prevotella intermedia*/  *nigrescens* (320)  *Fusobacterium nucleatum* (122)  *Parvimonas micra* (364)  *Streptococcus constellatus* (147)  *Aggregatibacter actinomycetemcomitans* (81)  Enteric rods/ pseudomonads (9)  *Enterococcus faecalis* (4)  *Staphylococcus aureus* (1) | Culture | AMX*, CLI*, DOX*, MTZ*, AMX+MTZ* | NA | NA |
| *Americas*  *(United States of America)* | Rams *et al.,* 2014b | Patients (50)  Severe periodontal disease | 31 – 76 years | *Streptococcus constellatus* (33)  *Streptococcus intermedius* (17) | Culture | AMX, AZI*, CIP*, CLI*, DOX*, MTZ* | NA | NA |
| *Americas (Colombia)* | Ardila et al., 2020 | Patients (76)  Aggressive periodontal disease | 26,1 (mean) | *Aggregatibacter*  *actinomycetemcomitans* (37)  *Porphyromonas gingivalis* (61)  *Tanerella forsythia* (43) | Culture | AMX*, AZI*, MTZ*, MOX | NA | NA |
| *Americas*  *(United States of America)* | Rams *et al.,* 2020 | Patients (88)  Severe periodontal disease | 35 – 83 years | *Porphyromonas gingivalis* (9)  *Tannerella forsythia* (47)  *Prevotella intermedia*/  *nigrescens* (80)  *Fusobacterium nucleatum* (73)  *Parvimonas micra* (88)  *Streptococcus constellatus* (9)  *Campylobacter rectus* (13) | Culture | AMX*, CLI*, DOX*, MTZ*, TIN* | NA | NA |
| *Americas*  *(Peru)* | Aguilar-Luis *et al*., 2021 | Patients (8)  Non-specific periodontal disease | NR | *Rothia dentocariosa*  *Eikenella corrodens*  *Granulicatella adiacens*  *Actinomyces naeslundii* | Culture | AMP*, AXL*, AZI*, CIP*, CLI*, CTZ*, DIC*, MTZ*, PEN*, TET* | NA | NA |
| *Americas*  *(Brazil)* | Ansiliero *et al.,* 2021 | Patients (18)  Non-specific periodontal disease | 13 – 67 years | *Citrobacter freundii*  *Enterobacter aerogenes Enterococcus avium*  *Escherichia coli*  *Klebsiella ozaenae*  *Pseudomonas aeruginosa*  *Pseudomonas fluorescens*  *Raoutella spp.*  *Staphylococcus aureus*  *Staphylococcus haemolyticus*  *Streptococcus pneumoniae*  *Streptococcus salivarius* | Culture | AMX*, AZI*, CEF*, CFM*, CLI*, CLR*, IMI*, TET* | NA | NA |
| *Americas (Dominican Republic)* | Collins *et al*., 2015 | Patients (77)  Aggressive periodontal disease/ chronic disease | 18 – 65 years | NA | Molecular technique | NA | *tet*, *tet*B, *tet*L, *tet*M, *tet*Q, *tet*O, *tet*W (TET) | NA |
| *Americas (Brazil)* | Almeida *et al.,* 2020 | Patients (110)  Non-specific periodontal disease/ healthy patients | 41,5 (mean) | NA | Molecular technique | NA | *aac* (fluroquinolones),  *bla*_TEM,_ *mecA* (ß-lactamase),  *erm* (ERY),  *pbp2b* (PEN) | NA |
| *Americas*  *(Mexico)* | Uribe-Garcia *et al.,* 2019 | Patients (268)  Non-specific periodontal disease | NR | *Staphylococcus aureus* (50) | Culture  Molecular technique | AMP*, CEF*, CFM*, CRX*, CTZ*, DIC*, ERY*, GEN*, LEV*, PEF*, PEN*, TET* |  | bbp (bone sialoprotein binding protein)  clfA, clfB (*S. aureus* binding to fibrinogen),  cna (*S. aureus* binding to collagen)  coa (coagulase enzyme),  ebps (elastin binding protein)  fnbA, fnbB (*S. aureus* binding to fibronectin),  map/eap (extracellular adhesion protein),  sdrC, sdrD, sdrE (sialoprotein and fibrinogen binding protein)  spa (staphylococcal protein A) |
| *Asia*  *(India)* | Dhotre *et al.,* 2015 | Patients (80)  Non-specific periodontal disease/ healthy patients | 49 (mean) | *Staphylococcus aureus* (1)  *Streptococcus mitis* (50)  *Streptococcus oralis* (45)  *Streptococcus sanguinis* (41)  *Streptococcus parasanguinis* (12)  *Streptococcus gordonii* (4)  *Streptococcus anginosus* (6)  *Streptococcus constellatus* (7)  *Streptococcus mutans* (30)  *Streptococcus hyointestinalis* (1)  *Streptococcus sinensis* (2)  *Streptococcus pluranimalium* (1)  *Streptococcus thoraltensis* (1)  *Streptococcus tigurinus* (1)  *Granulicatella adiacens* (21)  *Granulicatella elegans* (38) | Culture | AMP*, AZI*, CEM*, CFM*, CXE*, CLA*, CLI*, ERI*, LEV*, LIZ, OFX*, PEN*, QUI*, TET*, VAN | NA | NA |
| *Asia*  *(India)* | Bhardwaj *et al.,* 2017 | Patients (100)  Chronic periodontal disease | 18 – 75 years | Enterococci (46) | Culture | AMX*, CIP*, ERY*, GEN*, TEI*, VAN* | NA | NA |
| *Asia*  *(India)* | Bhat *et al.,* 2019 | Patients (40)  Severe periodontal disease | NR | *Aggregatibacter*  *actinomycetemcomitans* (40) | Culture | AMX*, AXL*, AZI*, CFZ*, CFM*, CXE*, CRX*, CLI*, DOX*, MTZ, MOX*, TET* | NA | NA |
| *Asia*  *(Pakistan)* | Irshad *et al.,* 2020 | Patients (45)  Non-specific periodontal disease | 45,4 + 7,5 (range) | *Prevotella intermedia/*  *nigrescens* (10)  *Porphyromonas gingivalis* (10)  *Actinobacillus actinomycetemcomitans* (6) | Culture | AMX*, AZI, MTZ*, TET* | NA | NA |
| *Asia*  *(China)* | Xie *et al.,* 2014 | Patients (41)  Non-specific periodontal disease | 47 (mean) | *Prevotella* genus (42)  Não-*Prevotella* anaeróbios (18) | Culture  Molecular technique | AMX*, CFX*, CED*, CLI*, DOX*, IMI, MTZ*, ROX* | *bla*_CfxA_ (ß-lactamase)  *erm(*F) (ERY),  *nim* (MTZ)  *tet*Q (TET) | NA |
| *Europe*  *(Spain)* | DeLaTorre *et al.,* 2017 | Patients (61)  Chronic periodontal disease | NR | *Candida* spp. (126) | Culture | AFB*, FLU*, ITR*, MIC*, NYS*, POS*, VOR* | NA | NA |
| *Europe*  *(United Kingdom)* | Akrivopoulou *et al*., 2017 | Patients (50)  Aggressive periodontal disease | NR | *Aggregatibacter*  *actinomycetemcomitans* (56) | Culture | AMX*, AXL*, CZD*, CIP, CLI*, MTZ*, PEN*, TET* | NA | NA |
| *Europe*  *(France)* | Ehrmann *et al.,* 2014 | Patients (42)  Non-specific periodontal disease/ healthy patients | NR | *Capnocytophaga* spp. (48) | Culture  Molecular technique | AMX*, AXL, CFM*, CZD*, CLI, ERY* | *bla*_CfxA_, *bla*_CSP-1_, (ß-lactamase)  *erm*(C)/ *erm*(F) (ERY) | NA |
| *Europe*  *(Spain)* | Arredondo *et al.,* 2019 | Patients (52)  Non-specific periodontal disease | 49,8 (range) | *Prevotella* spp. (100) | Culture  Molecular technique | AZI*, ERY | *erm*B*, erm*F (ERY) | NA |
| *Europe*  *(Spain)* | Arredondo *et al.,* 2020 | Patients (130)  Aggressive periodontal disease | 24 – 82 years | *Actinomyces* spp.;  *Alloprevotella* spp.;  *Anaerococcus* spp.;  *Bifidobacterium* spp.;  *Campylobacter* spp.;  *Capnocytophaga* spp.;  *Dialister* spp.;  *Eikenella* spp.;  *Escherichia* spp.;  *Fusobacterium* spp.;  *Klebsiella* spp.;  *Leptotrichia* spp.;  *Morganella* spp.;  *Olsenella* spp.;  *Peptostreptococcus* spp.;  *Prevotella* spp.;  *Pseudomonas* spp.;  *Rothia* spp.;  *Serratia* spp.;  *Staphylococcus* spp.;  *Streptococcus* spp.;  *Veillonella* spp. | Culture  Molecular technique | AMX*, KAN*, CFM*, CLR*, ERY*, STR*, TET* | *bla*_TEM_, *bla*_SHV_, *bla*_CfxA_, *bla*_CepA_, *bla*_CblA_ (ß-lactamase) | NA |
| Antimicrobials assessed: AFB - anfotericin B, AFX – amifloxacin, AMI – amikacin, AMX – amoxicillin, AXL – amoxicillin + clavulanic acid, AMP – ampicillin, AZI – azithromycin, CFZ – cefazolin, CEF – cefalotin, CEM – cefepime, CFX – cefixime, CFM – cefotaxime, CED – cefradine, CZD – ceftazidime, CXE – ceftriaxone, CRX – cefuroxime, CLR – chloramphenicol, CIP – ciprofloxacin, CLA – clarithromycin, CLI – clindamycin, CTZ – cotrimoxazole, DIC – dicloxacillin, DOX – doxycycline, ERY – erythromycin, FOS – fosfomycin, FLU – fluconazole, GEN – gentamicin, IMI – imipenem, ITR – itraconazole, KAN - kanamycin, LEV – levofloxacin, LIZ – linezolid, MOX – moxifloxacin, MTZ – metronidazole, MIC – miconazole, NIT – nitrofurantoin, NYS – nystatin, OFX – ofloxacin, PEF – pefloxacin, PEN – penicillin, POS – posaconazole, QUI – quinupristin/ dalfopristin, ROX – roxithromycin, SPI – spiramycin, STR – Streptomycin, TEI – teicoplanin, TET – tetracycline, TGC – tigecycline, TIN – tinidazole, VAN – vancomycin, VOR – voriconazole.  * – resistance reported  NA – not assessed, NR – not reported. | | | | | | | | |
