# Supplementary material for: Antimicrobial resistance of microorganisms present in periodontal diseases: A systematic review and meta-analysis
Source: Front Microbiol. 2022 Oct 3;13:961986. doi: 10.3389/fmicb.2022.961986 (PMC9574196; doi:10.3389/fmicb.2022.961986)
Supplement: Supplementary file 4 [file Data_Sheet_4.docx]

**Supplementary Material 4** – Metanalyses of the most cited antimicrobials.

Amoxicillin

Amoxicillin + clavulanic acid

Ampicillin

Azithromycin

Cefotaxime

Ciprofloxacin

Clindamycin

Doxycycline

Metronidazole

Penicillin

Tetracycline
