# Supplementary material for: Antimicrobial resistance of microorganisms present in periodontal diseases: A systematic review and meta-analysis
Source: Front Microbiol. 2022 Oct 3;13:961986. doi: 10.3389/fmicb.2022.961986 (PMC9574196; doi:10.3389/fmicb.2022.961986)

Amoxicillin

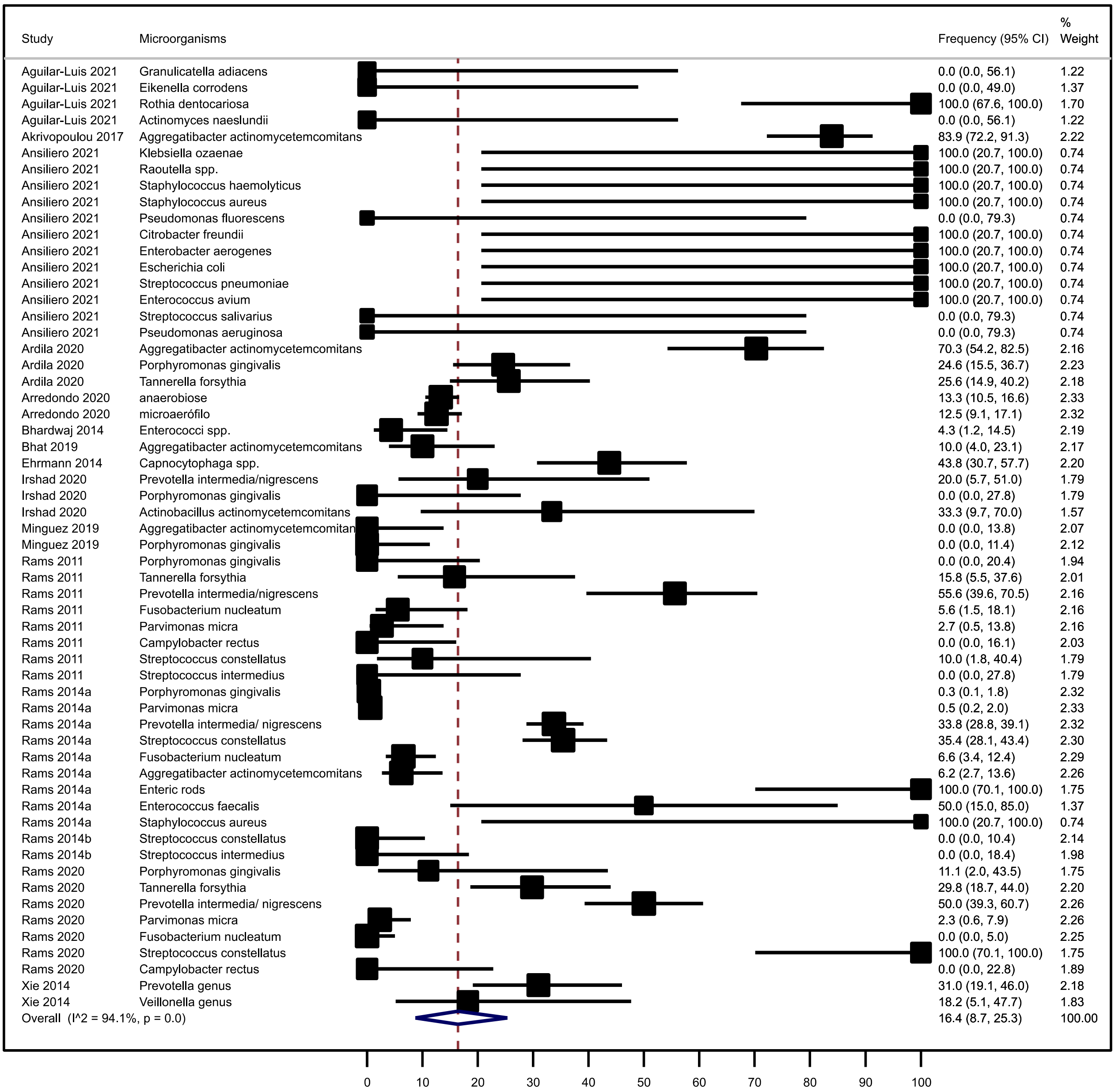

# Amoxicillin + clavulanic acid

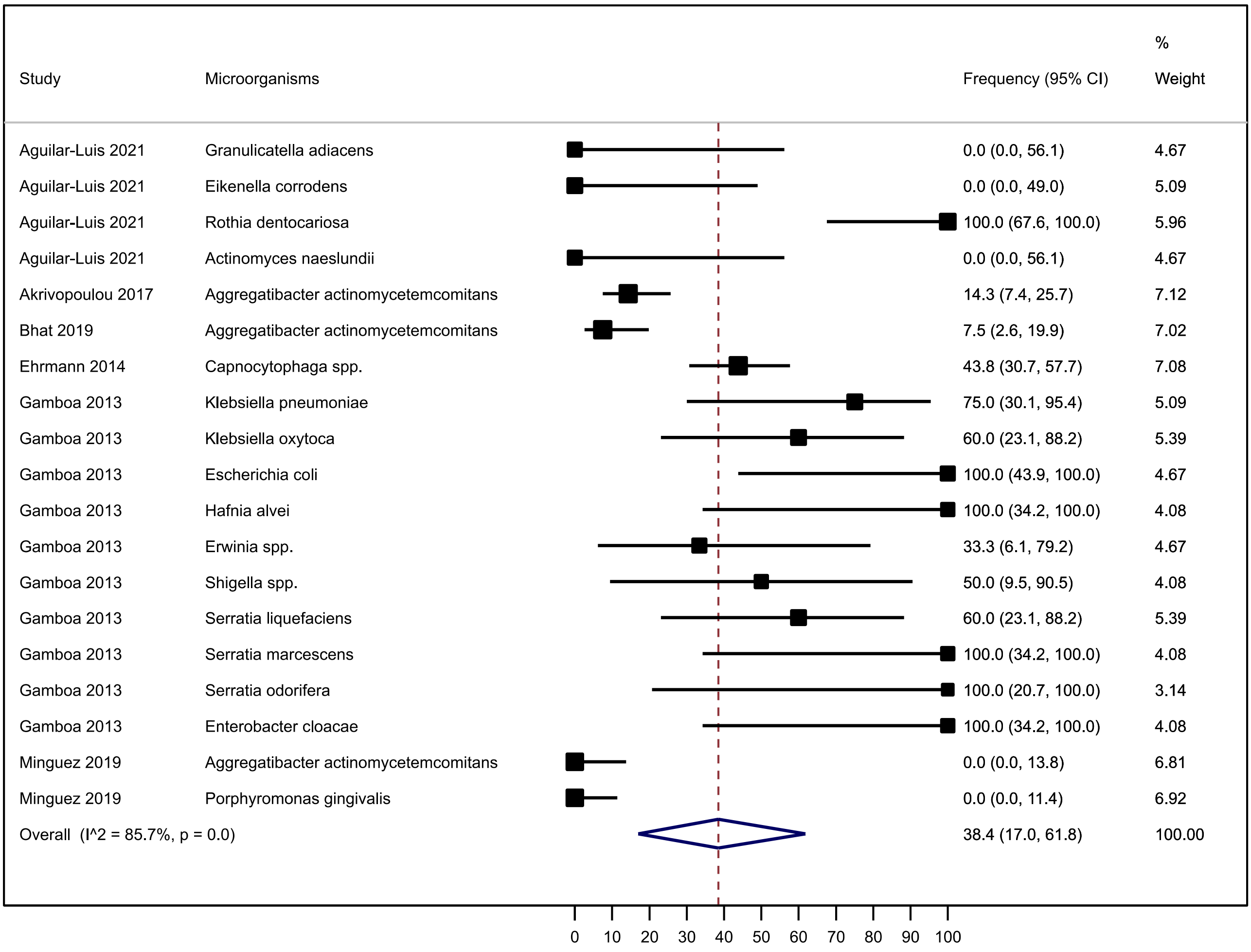

Ampicillin

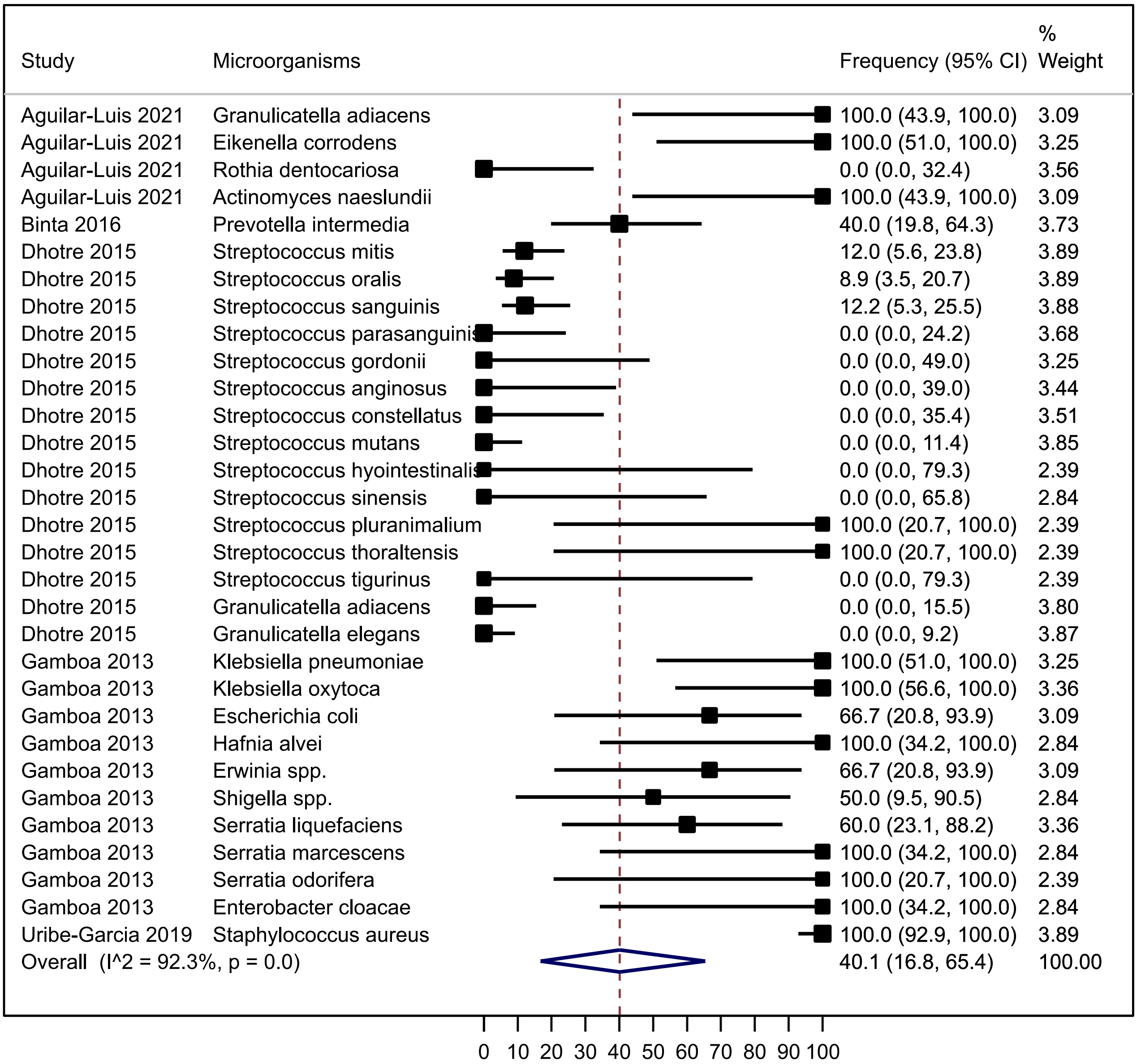
Study
Microorganisms
Frequency (95% CI)
% Weight
Aguilar-Luis 2021 Granulicatella adiacens 100.0 (43.9, 100.0) 3.09
Aguilar-Luis 2021 Eikenella corrodens 100.0 (51.0, 100.0) 3.25
Aguilar-Luis 2021 Rothia dentocariosa 0.0 (0.0, 32.4) 3.56
Aguilar-Luis 2021 Actinomyces naeslundii 100.0 (43.9, 100.0) 3.09
Binta 2016 Prevotella intermedia 40.0 (19.8, 64.3) 3.73
Dhotre 2015 Streptococcus mitis 12.0 (5.6, 23.8) 3.89
Dhotre 2015 Streptococcus oralis 8.9 (3.5, 20.7) 3.89
Dhotre 2015 Streptococcus sanguinis 12.2 (5.3, 25.5) 3.88
Dhotre 2015 Streptococcus parasanguinis 0.0 (0.0, 24.2) 3.68
Dhotre 2015 Streptococcus gordonii 0.0 (0.0, 49.0) 3.25
Dhotre 2015 Streptococcus anginosus 0.0 (0.0, 39.0) 3.44
Dhotre 2015 Streptococcus constellatus 0.0 (0.0, 35.4) 3.51
Dhotre 2015 Streptococcus mutans 0.0 (0.0, 11.4) 3.85
Dhotre 2015 Streptococcus hyointestinalis 0.0 (0.0, 79.3) 2.39
Dhotre 2015 Streptococcus sinensis 0.0 (0.0, 65.8) 2.84
Dhotre 2015 Streptococcus pluranimalium 100.0 (20.7, 100.0) 2.39
Dhotre 2015 Streptococcus thoraltensis 100.0 (20.7, 100.0) 2.39
Dhotre 2015 Streptococcus tigurinus 0.0 (0.0, 79.3) 2.39
Dhotre 2015 Granulicatella adiacens 0.0 (0.0, 15.5) 3.80
Dhotre 2015 Granulicatella elegans 0.0 (0.0, 9.2) 3.87
Gamboa 2013 Klebsiella pneumoniae 100.0 (51.0, 100.0) 3.25
Gamboa 2013 Klebsiella oxytoca 100.0 (56.6, 100.0) 3.36
Gamboa 2013 Escherichia coli 66.7 (20.8, 93.9) 3.09
Gamboa 2013 Hafnia alvei 100.0 (34.2, 100.0) 2.84
Gamboa 2013 Erwinia spp. 66.7 (20.8, 93.9) 3.09
Gamboa 2013 Shigella spp. 50.0 (9.5, 90.5) 2.84
Gamboa 2013 Serratia liquefaciens 60.0 (23.1, 88.2) 3.36
Gamboa 2013 Serratia marcescens 100.0 (34.2, 100.0) 2.84
Gamboa 2013 Serratia odorifera 100.0 (20.7, 100.0) 2.39
Gamboa 2013 Enterobacter cloacae 100.0 (34.2, 100.0) 2.84
Uribe-Garcia 2019 Staphylococcus aureus 100.0 (92.9, 100.0) 3.89
Overall (I^2 = 92.3%, p = 0.0) 40.1 (16.8, 65.4) 100.00
0 10 20 30 40 50 60 70 80 90 100

Azithromycin

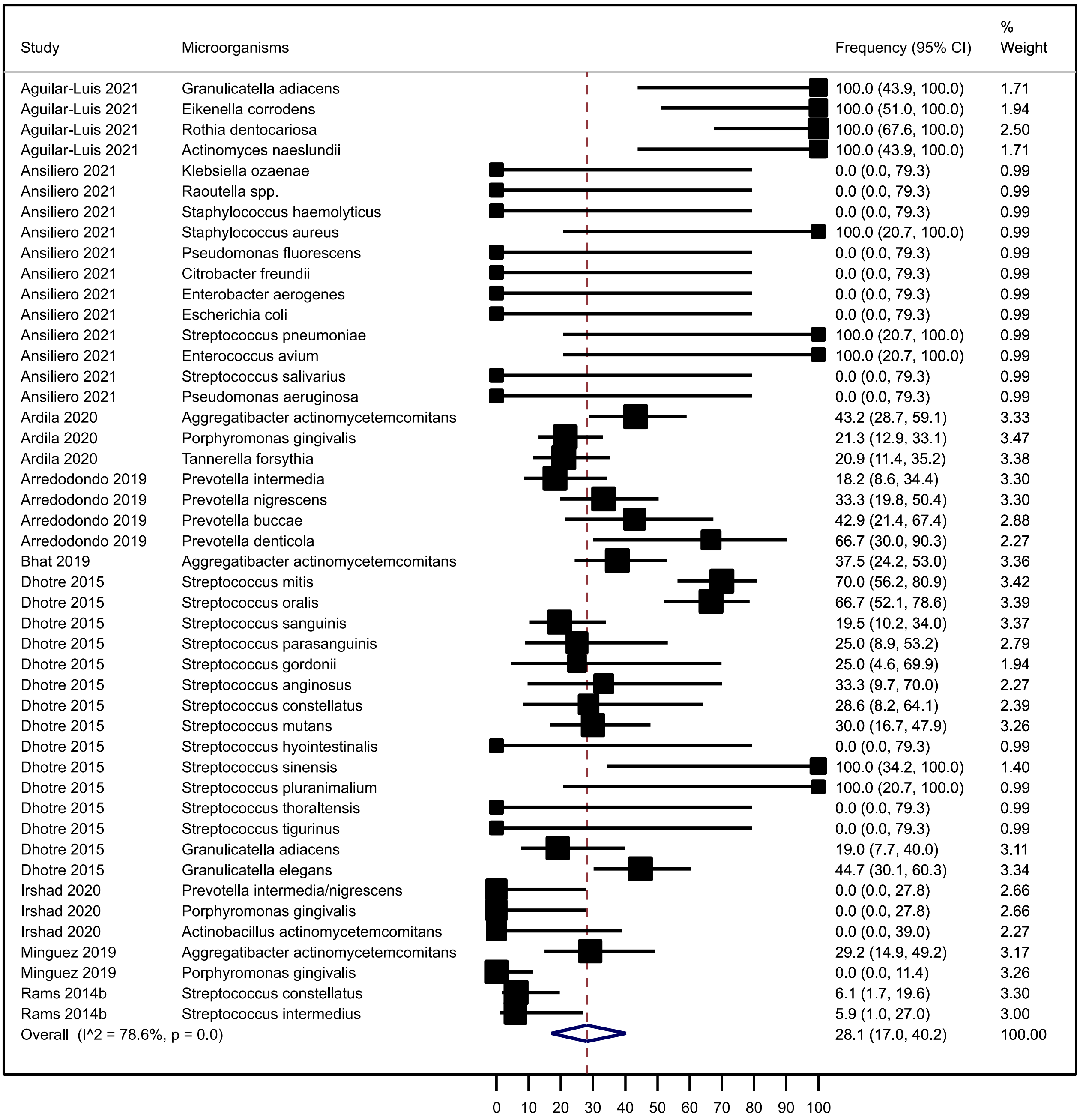

Cefotaxime

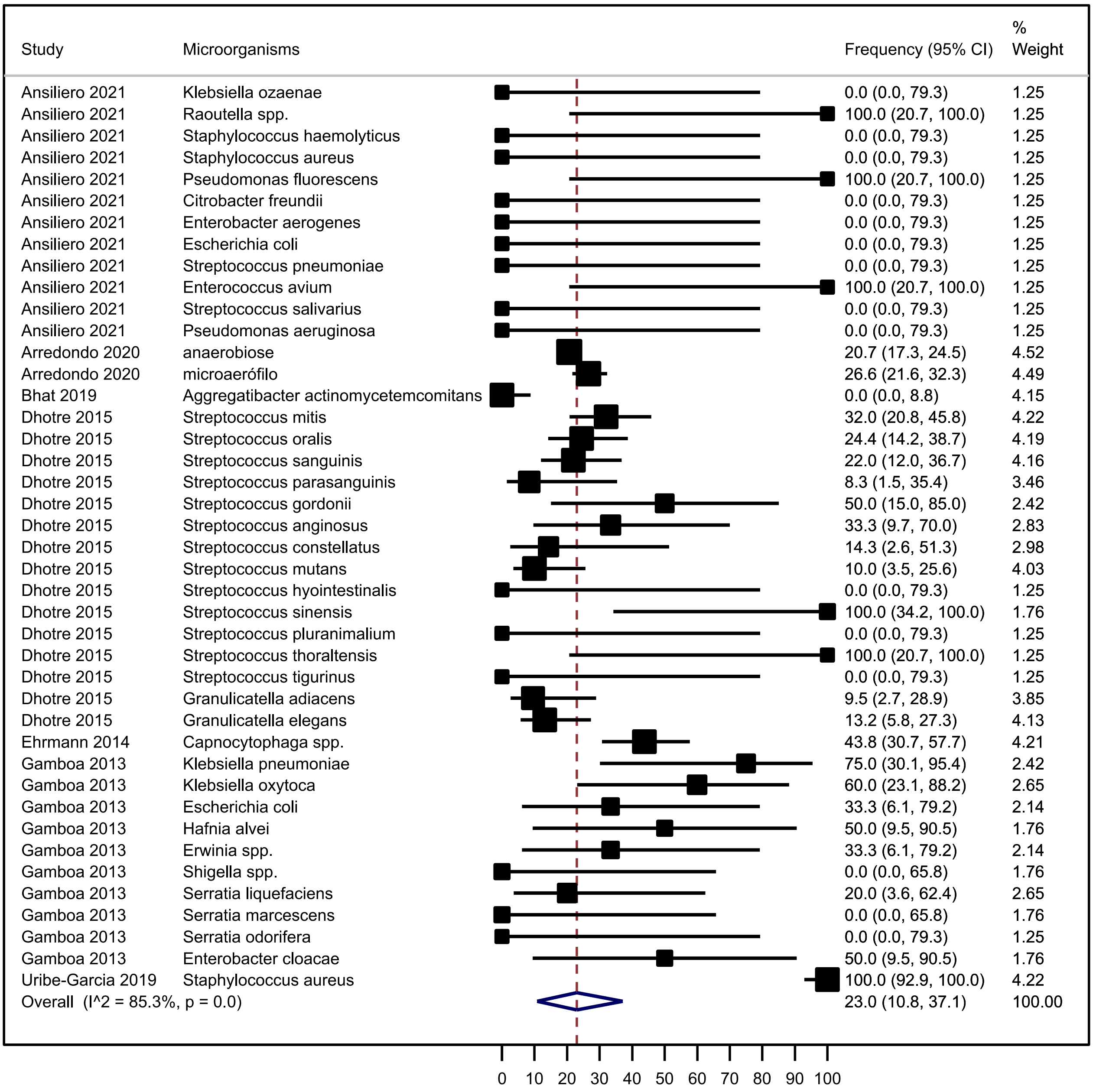

Study | Microorganisms | Frequency (95% CI) | % Weight
Ansiliero 2021 | Klebsiella ozaenae | 0.0 (0.0, 79.3) | 1.25
Ansiliero 2021 | Raoutella spp. | 100.0 (20.7, 100.0) | 1.25
Ansiliero 2021 | Staphylococcus haemolyticus | 0.0 (0.0, 79.3) | 1.25
Ansiliero 2021 | Staphylococcus aureus | 0.0 (0.0, 79.3) | 1.25
Ansiliero 2021 | Pseudomonas fluorescens | 100.0 (20.7, 100.0) | 1.25
Ansiliero 2021 | Citrobacter freundii | 0.0 (0.0, 79.3) | 1.25
Ansiliero 2021 | Enterobacter aerogenes | 0.0 (0.0, 79.3) | 1.25
Ansiliero 2021 | Escherichia coli | 0.0 (0.0, 79.3) | 1.25
Ansiliero 2021 | Streptococcus pneumoniae | 0.0 (0.0, 79.3) | 1.25
Ansiliero 2021 | Enterococcus avium | 100.0 (20.7, 100.0) | 1.25
Ansiliero 2021 | Streptococcus salivarius | 0.0 (0.0, 79.3) | 1.25
Ansiliero 2021 | Pseudomonas aeruginosa | 0.0 (0.0, 79.3) | 1.25
Arredondo 2020 | anaerobiose | 20.7 (17.3, 24.5) | 4.52
Arredondo 2020 | microaerófilo | 26.6 (21.6, 32.3) | 4.49
Bhat 2019 | Aggregatibacter actinomycetemcomitans | 0.0 (0.0, 8.8) | 4.15
Dhotre 2015 | Streptococcus mitis | 32.0 (20.8, 45.8) | 4.22
Dhotre 2015 | Streptococcus oralis | 24.4 (14.2, 38.7) | 4.19
Dhotre 2015 | Streptococcus sanguinis | 22.0 (12.0, 36.7) | 4.16
Dhotre 2015 | Streptococcus parasanguinis | 8.3 (1.5, 35.4) | 3.46
Dhotre 2015 | Streptococcus gordonii | 50.0 (15.0, 85.0) | 2.42
Dhotre 2015 | Streptococcus anginosus | 33.3 (9.7, 70.0) | 2.83
Dhotre 2015 | Streptococcus constellatus | 14.3 (2.6, 51.3) | 2.98
Dhotre 2015 | Streptococcus mutans | 10.0 (3.5, 25.6) | 4.03
Dhotre 2015 | Streptococcus hyointestinalis | 0.0 (0.0, 79.3) | 1.25
Dhotre 2015 | Streptococcus sinensis | 100.0 (34.2, 100.0) | 1.76
Dhotre 2015 | Streptococcus pluranimalium | 0.0 (0.0, 79.3) | 1.25
Dhotre 2015 | Streptococcus thoraltensis | 100.0 (20.7, 100.0) | 1.25
Dhotre 2015 | Streptococcus tigurinus | 0.0 (0.0, 79.3) | 1.25
Dhotre 2015 | Granulicatella adiacens | 9.5 (2.7, 28.9) | 3.85
Dhotre 2015 | Granulicatella elegans | 13.2 (5.8, 27.3) | 4.13
Ehrmann 2014 | Capnocytophaga spp. | 43.8 (30.7, 57.7) | 4.21
Gamboa 2013 | Klebsiella pneumoniae | 75.0 (30.1, 95.4) | 2.42
Gamboa 2013 | Klebsiella oxytoca | 60.0 (23.1, 88.2) | 2.65
Gamboa 2013 | Escherichia coli | 33.3 (6.1, 79.2) | 2.14
Gamboa 2013 | Hafnia alvei | 50.0 (9.5, 90.5) | 1.76
Gamboa 2013 | Erwinia spp. | 33.3 (6.1, 79.2) | 2.14
Gamboa 2013 | Shigella spp. | 0.0 (0.0, 65.8) | 1.76
Gamboa 2013 | Serratia liquefaciens | 20.0 (3.6, 62.4) | 2.65
Gamboa 2013 | Serratia marcescens | 0.0 (0.0, 65.8) | 1.76
Gamboa 2013 | Serratia odorifera | 0.0 (0.0, 79.3) | 1.25
Gamboa 2013 | Enterobacter cloacae | 50.0 (9.5, 90.5) | 1.76
Uribe-Garcia 2019 | Staphylococcus aureus | 100.0 (92.9, 100.0) | 4.22
Overall (I^2 = 85.3%, p = 0.0) | 23.0 (10.8, 37.1) | 100.00
0 10 20 30 40 50 60 70 80 90 100

# Ciprofloxacin

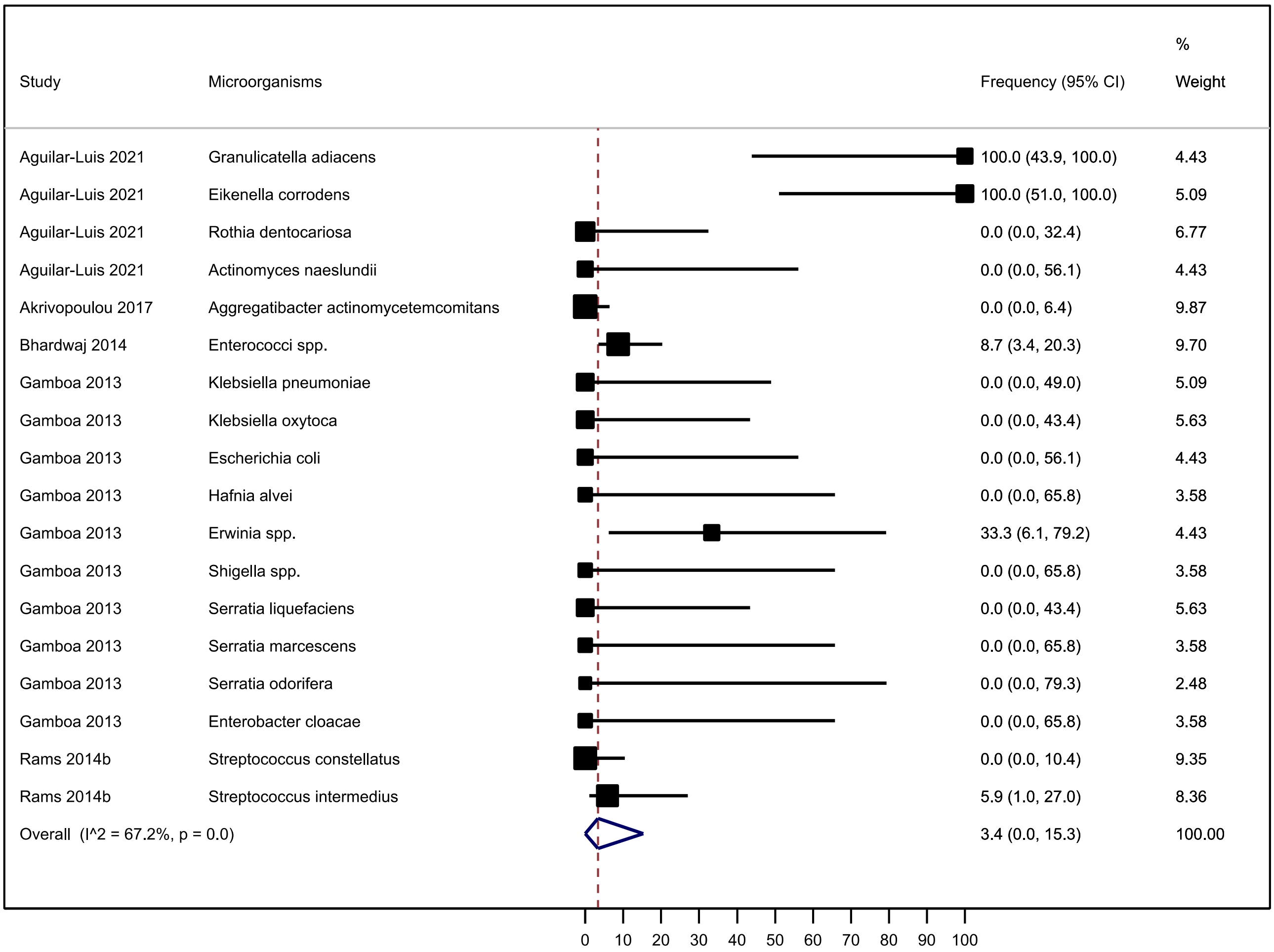

Clindamycin

| Study | Microorganisms | Frequency (95% CI) | % Weight |
|---|---|---|---|
| Aguilar-Luis 2021 | Granulicatella adiacens | 100.0 (43.9, 100.0) | 1.64 |
| Aguilar-Luis 2021 | Eikenella corrodens | 100.0 (51.0, 100.0) | 1.77 |
| Aguilar-Luis 2021 | Rothia dentocariosa | 100.0 (67.6, 100.0) | 2.03 |
| Aguilar-Luis 2021 | Actinomyces naeslundii | 100.0 (43.9, 100.0) | 1.64 |
| Akrivopoulou 2017 | Aggregatibacter actinomycetemcomitans | 87.5 (76.4, 93.8) | 2.37 |
| Ansiliero 2021 | Klebsiella ozaenae | 0.0 (0.0, 79.3) | 1.14 |
| Ansiliero 2021 | Raoutella spp. | 0.0 (0.0, 79.3) | 1.14 |
| Ansiliero 2021 | Staphylococcus haemolyticus | 0.0 (0.0, 79.3) | 1.14 |
| Ansiliero 2021 | Staphylococcus aureus | 0.0 (0.0, 79.3) | 1.14 |
| Ansiliero 2021 | Pseudomonas fluorescens | 0.0 (0.0, 79.3) | 1.14 |
| Ansiliero 2021 | Citrobacter freundii | 0.0 (0.0, 79.3) | 1.14 |
| Ansiliero 2021 | Enterobacter aerogenes | 0.0 (0.0, 79.3) | 1.14 |
| Ansiliero 2021 | Escherichia coli | 0.0 (0.0, 79.3) | 1.14 |
| Ansiliero 2021 | Streptococcus pneumoniae | 100.0 (20.7, 100.0) | 1.14 |
| Ansiliero 2021 | Enterococcus avium | 100.0 (20.7, 100.0) | 1.14 |
| Ansiliero 2021 | Streptococcus salivarius | 0.0 (0.0, 79.3) | 1.14 |
| Ansiliero 2021 | Pseudomonas aeruginosa | 0.0 (0.0, 79.3) | 1.14 |
| Bhat 2019 | Aggregatibacter actinomycetemcomitans | 62.5 (47.0, 75.8) | 2.34 |
| Dhotre 2015 | Streptococcus mitis | 46.0 (33.0, 59.6) | 2.36 |
| Dhotre 2015 | Streptococcus oralis | 46.7 (32.9, 60.9) | 2.35 |
| Dhotre 2015 | Streptococcus sanguinis | 29.3 (17.6, 44.5) | 2.34 |
| Dhotre 2015 | Streptococcus parasanguinis | 16.7 (4.7, 44.8) | 2.15 |
| Dhotre 2015 | Streptococcus gordonii | 25.0 (4.6, 69.9) | 1.77 |
| Dhotre 2015 | Streptococcus anginosus | 16.7 (3.0, 56.4) | 1.93 |
| Dhotre 2015 | Streptococcus constellatus | 28.6 (8.2, 64.1) | 1.99 |
| Dhotre 2015 | Streptococcus mutans | 36.7 (21.9, 54.5) | 2.31 |
| Dhotre 2015 | Streptococcus hyointestinalis | 0.0 (0.0, 79.3) | 1.14 |
| Dhotre 2015 | Streptococcus sinensis | 0.0 (0.0, 65.8) | 1.45 |
| Dhotre 2015 | Streptococcus pluranimalium | 100.0 (20.7, 100.0) | 1.14 |
| Dhotre 2015 | Streptococcus thoraltensis | 0.0 (0.0, 79.3) | 1.14 |
| Dhotre 2015 | Streptococcus tigurinus | 0.0 (0.0, 79.3) | 1.14 |
| Dhotre 2015 | Granulicatella adiacens | 28.6 (13.8, 50.0) | 2.26 |
| Dhotre 2015 | Granulicatella elegans | 36.8 (23.4, 52.7) | 2.34 |
| Ehrmann 2014 | Capnocytophaga spp. | 29.2 (18.2, 43.2) | 2.36 |
| Rams 2014a | Porphyromonas gingivalis | 0.6 (0.2, 2.3) | 2.43 |
| Rams 2014a | Parvimonas micra | 1.4 (0.6, 3.2) | 2.43 |
| Rams 2014a | Prevotella intermedia/ nigrescens | 3.8 (2.2, 6.4) | 2.43 |
| Rams 2014a | Streptococcus constellatus | 6.1 (3.3, 11.2) | 2.41 |
| Rams 2014a | Fusobacterium nucleatum | 0.0 (0.0, 3.1) | 2.41 |
| Rams 2014a | Aggregatibacter actinomycetemcomitans | 93.8 (86.4, 97.3) | 2.39 |
| Rams 2014a | Enteric rods | 100.0 (70.1, 100.0) | 2.07 |
| Rams 2014a | Enterococcus faecalis | 75.0 (30.1, 95.4) | 1.77 |
| Rams 2014a | Staphylococcus aureus | 100.0 (20.7, 100.0) | 1.14 |
| Rams 2014b | Streptococcus constellatus | 6.1 (1.7, 19.6) | 2.32 |
| Rams 2014b | Streptococcus intermedius | 0.0 (0.0, 18.4) | 2.22 |
| Rams 2020 | Porphyromonas gingivalis | 22.2 (6.3, 54.7) | 2.07 |
| Rams 2020 | Tannerella forsythia | 53.2 (39.2, 66.7) | 2.36 |
| Rams 2020 | Prevotella intermedia/ nigrescens | 58.7 (47.8, 68.9) | 2.39 |
| Rams 2020 | Parvimonas micra | 54.5 (44.2, 64.5) | 2.39 |
| Rams 2020 | Fusobacterium nucleatum | 0.0 (0.0, 5.0) | 2.38 |
| Rams 2020 | Streptococcus constellatus | 0.0 (0.0, 29.9) | 2.07 |
| Rams 2020 | Campylobacter rectus | 0.0 (0.0, 22.8) | 2.17 |
| Xie 2014 | Prevotella genus | 38.1 (25.0, 53.2) | 2.35 |
| Xie 2014 | Veillonella genus | 27.3 (9.7, 56.6) | 2.13 |
| Overall (I^2 = 95.8%, p = 0.0) | | 28.4 (15.0, 43.5) | 100.00 |

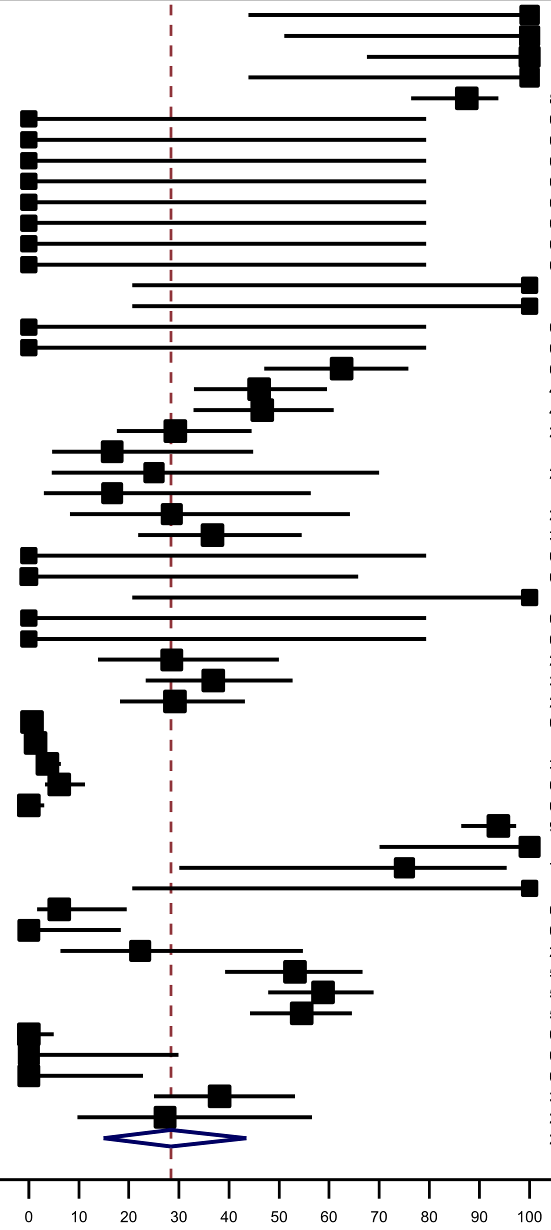

Doxycycline

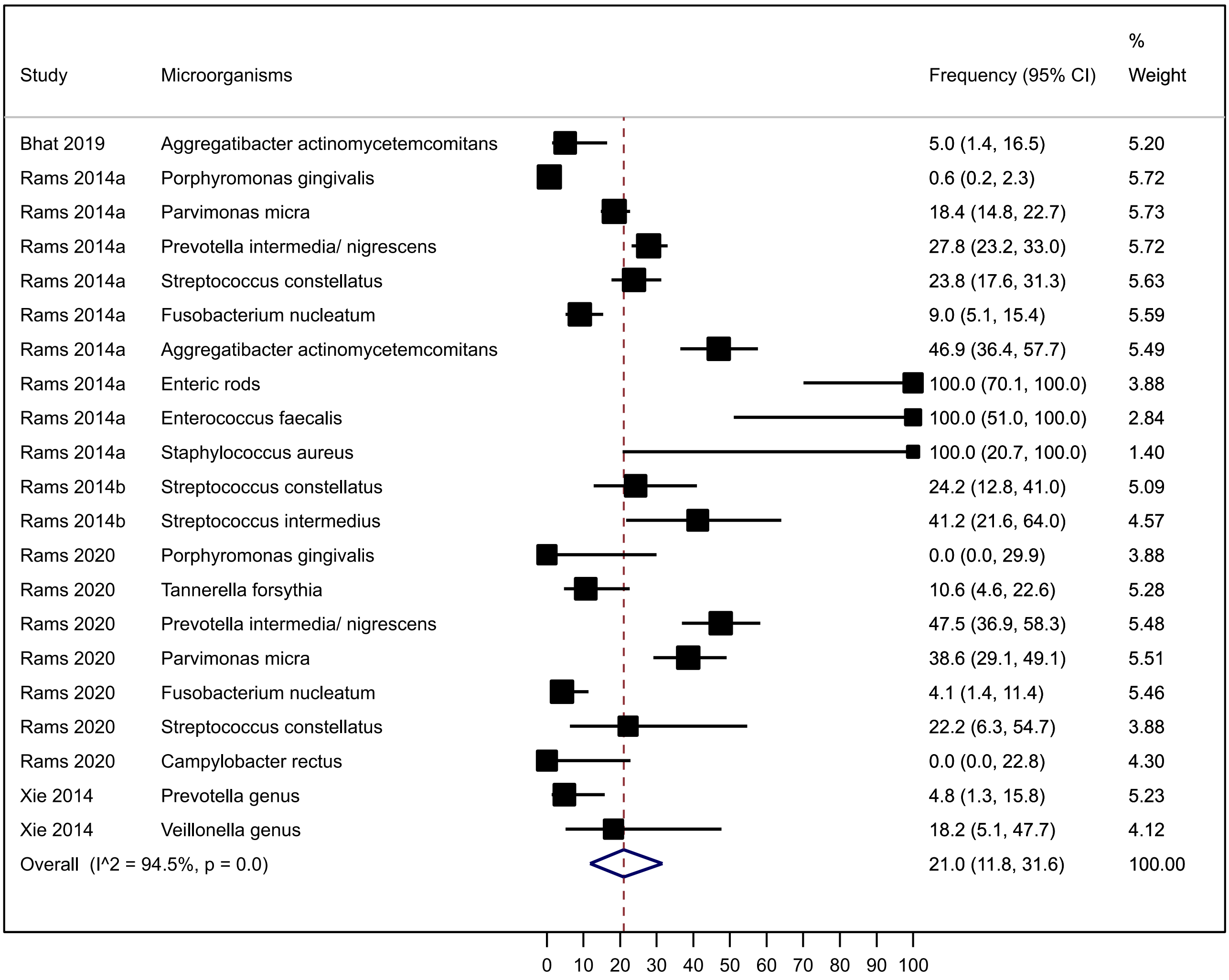

Metronidazole

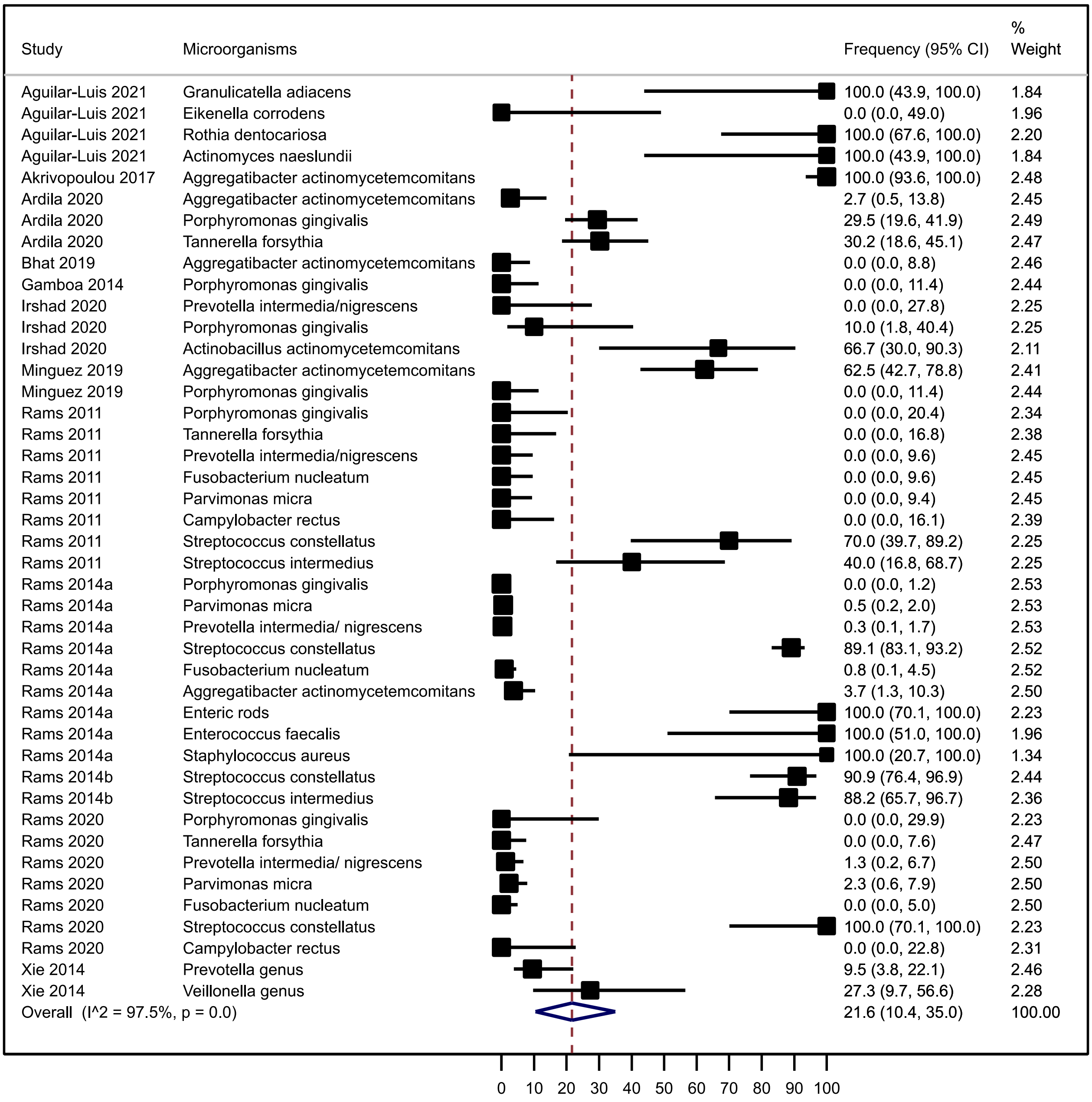

Penicillin

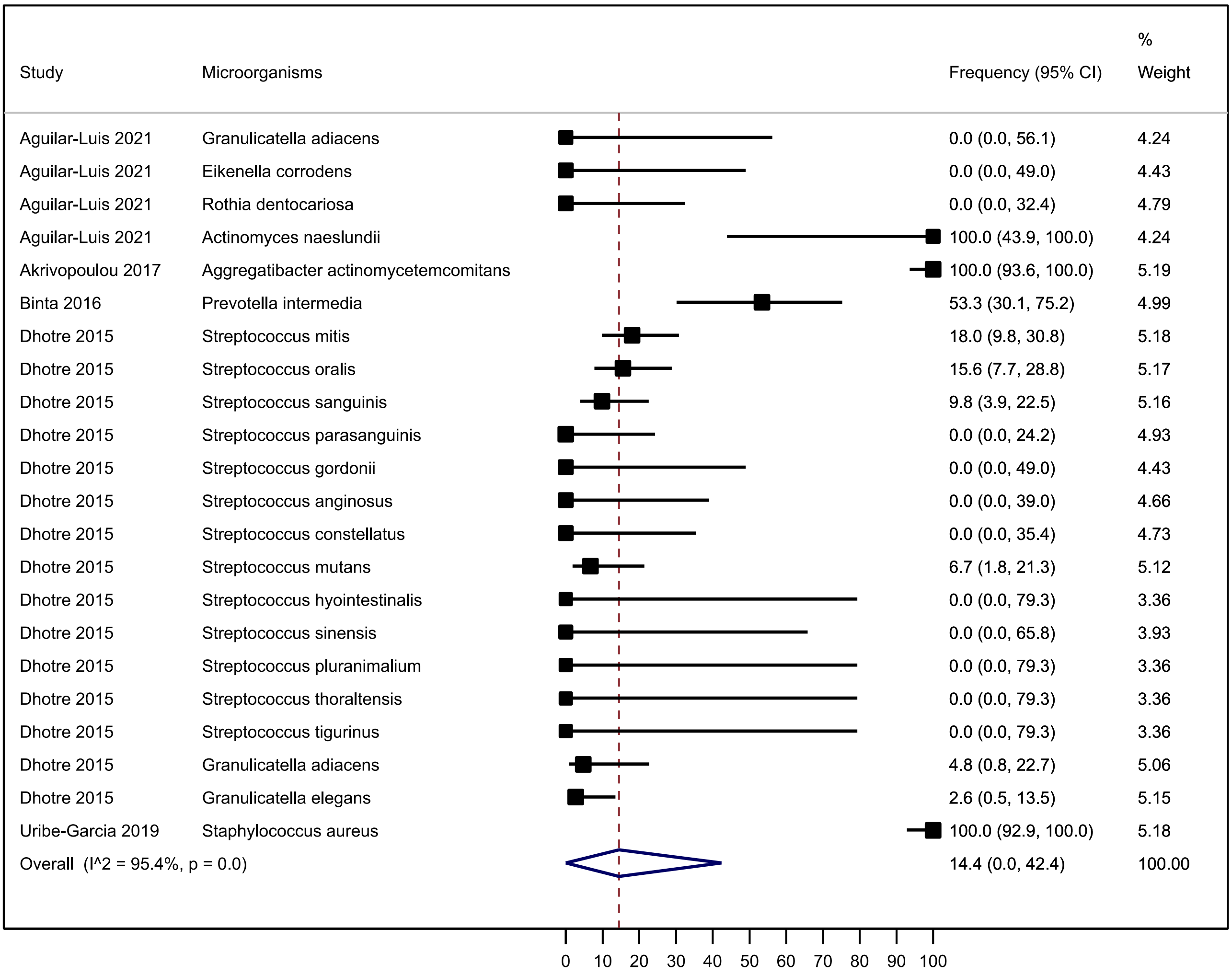
Study
Microorganisms
Frequency (95% CI)
% Weight
Aguilar-Luis 2021 Granulicatella adiacens 0.0 (0.0, 56.1) 4.24
Aguilar-Luis 2021 Eikenella corrodens 0.0 (0.0, 49.0) 4.43
Aguilar-Luis 2021 Rothia dentocariosa 0.0 (0.0, 32.4) 4.79
Aguilar-Luis 2021 Actinomyces naeslundii 100.0 (43.9, 100.0) 4.24
Akrivopoulou 2017 Aggregatibacter actinomycetemcomitans 100.0 (93.6, 100.0) 5.19
Binta 2016 Prevotella intermedia 53.3 (30.1, 75.2) 4.99
Dhotre 2015 Streptococcus mitis 18.0 (9.8, 30.8) 5.18
Dhotre 2015 Streptococcus oralis 15.6 (7.7, 28.8) 5.17
Dhotre 2015 Streptococcus sanguinis 9.8 (3.9, 22.5) 5.16
Dhotre 2015 Streptococcus parasanguinis 0.0 (0.0, 24.2) 4.93
Dhotre 2015 Streptococcus gordonii 0.0 (0.0, 49.0) 4.43
Dhotre 2015 Streptococcus anginosus 0.0 (0.0, 39.0) 4.66
Dhotre 2015 Streptococcus constellatus 0.0 (0.0, 35.4) 4.73
Dhotre 2015 Streptococcus mutans 6.7 (1.8, 21.3) 5.12
Dhotre 2015 Streptococcus hyointestinalis 0.0 (0.0, 79.3) 3.36
Dhotre 2015 Streptococcus sinensis 0.0 (0.0, 65.8) 3.93
Dhotre 2015 Streptococcus pluranimalium 0.0 (0.0, 79.3) 3.36
Dhotre 2015 Streptococcus thoraltensis 0.0 (0.0, 79.3) 3.36
Dhotre 2015 Streptococcus tigurinus 0.0 (0.0, 79.3) 3.36
Dhotre 2015 Granulicatella adiacens 4.8 (0.8, 22.7) 5.06
Dhotre 2015 Granulicatella elegans 2.6 (0.5, 13.5) 5.15
Uribe-Garcia 2019 Staphylococcus aureus 100.0 (92.9, 100.0) 5.18
Overall (I^2 = 95.4%, p = 0.0) 14.4 (0.0, 42.4) 100.00
0 10 20 30 40 50 60 70 80 90 100

Tetracycline

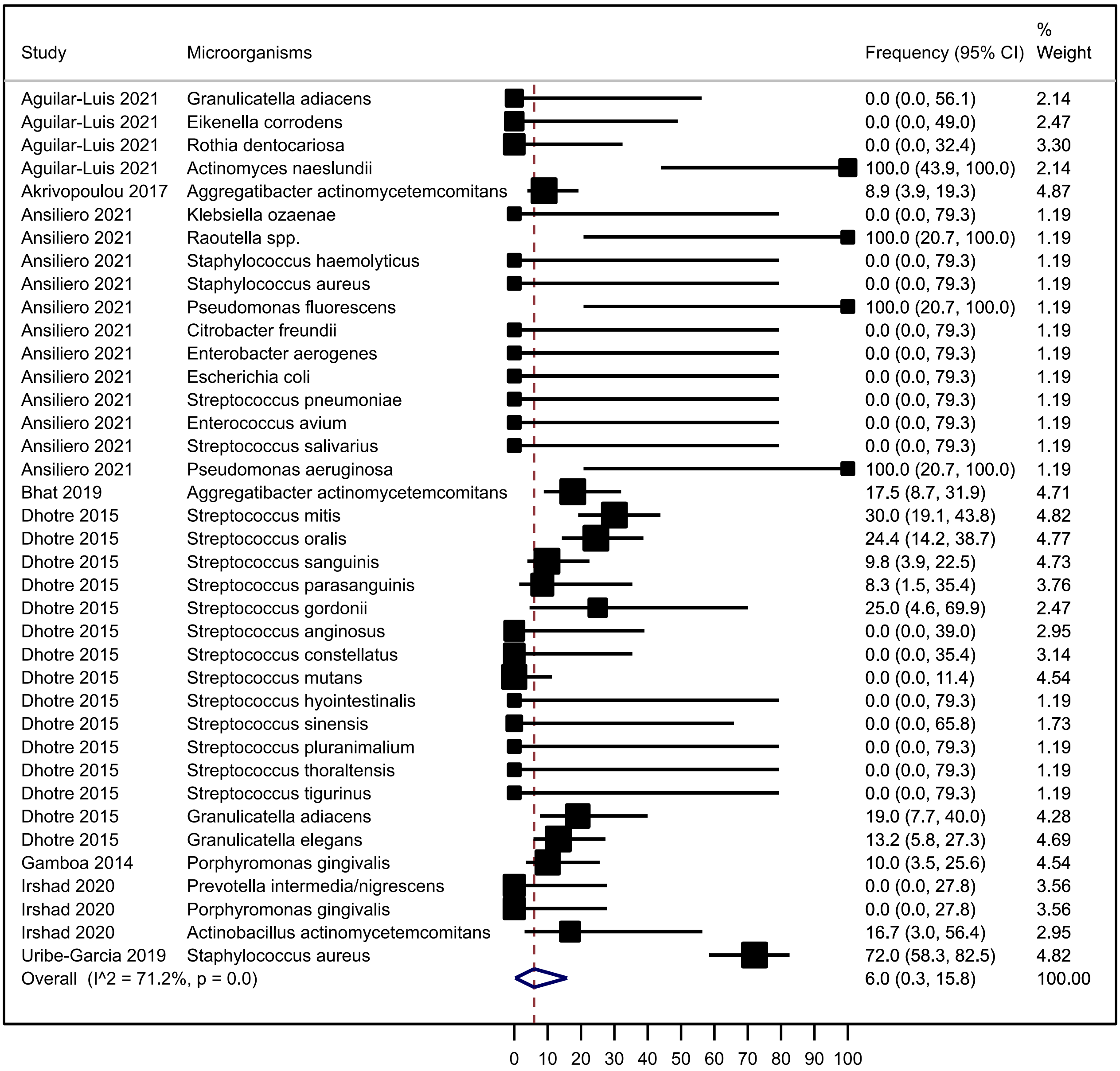

Supplement: Supplementary file 5 [file Image_1.pdf]
